# Healthcare worker fatigue during COVID-19, SARS, and MERS: a meta-analysis

**DOI:** 10.1093/occmed/kqag015

**Published:** 2026-03-07

**Authors:** K Poole-Wright, H Woodhall, T Chalder

**Affiliations:** Department of Psychological Medicine, Institute of Psychiatry, Psychology and Neuroscience, King’s College London, London SE5 8AB, UK; Department of Psychological Medicine, Institute of Psychiatry, Psychology and Neuroscience, King’s College London, London SE5 8AB, UK; Department of Psychological Medicine, Institute of Psychiatry, Psychology and Neuroscience, King’s College London, London SE5 8AB, UK

## Abstract

**Background:**

The physical and psychological impact of caring for patients during a coronavirus public health emergency had adverse effects on healthcare workers (HCW), including fatigue.

**Aims:**

To examine the prevalence of fatigue among HCW during severe acute respiratory syndrome (SARS), Middle East respiratory syndrome (MERS) or Coronavirus disease 19 (COVID-19) and identify associated risk and protective factors.

**Methods:**

Systematic searches of Embase, PsycINFO, Ovid-MEDLINE, CINAHL, HMIC and the Cochrane Library were conducted to July 2024. Inclusion criteria were English-language quantitative reports of fatigue in HCW during COVID-19, SARS and MERS. Random-effects meta-analyses were used to estimate pooled prevalence. Subgroup analyses examined fatigue by role, frontline status and personal protective equipment (PPE).

**Results:**

Eighty-eight articles (*n* = 74 914) met our inclusion criteria; 32 were eligible for meta-analysis. The pooled prevalence of fatigue was 55% (95% CI 46–65%, *k* = 32). Mental fatigue was reported by 58% (95% CI 17–90%, *k* = 4), while 53% (95% CI 38–67%, *k* = 11) experienced fatigue related to PPE use. No significant differences were observed between doctors and nurses (*P* = 0.327) or frontline and non-frontline staff (*P* = 0.103). Risk factors included stress, anxiety, depressive symptoms, workload and extended working hours, while resilience, self-efficacy and sufficient rest were protective. Substantial heterogeneity (*I*^2^ ∼99%) and reliance on cross-sectional designs limited causal inference.

**Conclusions:**

Our study indicated that over half of HCW reported fatigue and highlighted its multifactorial nature. Organizational-level interventions, such as optimized shift patterns, mandated rest breaks and psychological support are essential to mitigate fatigue, safeguard wellbeing and ensure safe healthcare provision.

Key learning points **What is already known about this subject:**

- Fatigue is a common problem among healthcare workers and is associated with several negative effects, including lower wellbeing and performance-related outcomes.
- COVID-19, severe acute respiratory syndrome, and Middle East respiratory syndrome greatly increased work demands and concomitant stress in frontline and non-frontline staff, with effects on increased fatigue.

**What this study adds:**

- Highlights that more than half of healthcare workers experienced physical and mental fatigue while working during a coronavirus pandemic or epidemic.
- Integrates and analyses current evidence for a range of associations with fatigue, which indicate that some organizational structures and individual differences impact the level of fatigue experienced.

**What impact this may have on practice or policy:**

- The current findings may inform hospital managers on who is most at risk for fatigue and where the risks are greatest.
- Organizational structures designed to manage and prevent fatigue could be implemented to provide enough rest, improve resilience and coping, and provide psychological support.

## INTRODUCTION

The COVID-19 pandemic presented unprecedented challenges to healthcare systems and healthcare providers worldwide. Healthcare professionals were tasked with treating potentially life-threatening symptoms while tackling the burden of care from large numbers of patients, often under conditions of uncertainty, resource scarcity and high personal risk. Alongside clinical demands, healthcare workers (HCW) faced stigma, social isolation and fears of being infected or infecting their family members [1]. These factors, combined with the psychological impact of caring for critically ill patients, had a deleterious effect on those working on the frontline [2,3]. Among the negative effects was fatigue, defined as ‘tiredness, a lack of energy and weakness, impeding participation in daily activities’ [4], associated with impairments in cognitive functioning, work performance and increased risk of errors [5]. Fatigue among HCW not only threatens individual health but impacts patient safety and healthcare resilience [6]. Fatigued staff are more likely to experience lapses in concentration, attention and reduced decision-making abilities [7], all of which increase the risk for medical errors [8]. In parallel, fatigue contributes to absenteeism, reduced motivation and higher turnover [9,10], weakening healthcare systems’ ability to respond effectively. Pandemic conditions, which increased workloads and psychological stressors, intensified these risks [11].

Previous studies provide evidence of this burden. During COVID-19, prevalence estimates for HCW fatigue ranged from 13% to 96% [3,12], while during the severe acute respiratory syndrome (SARS) epidemic up to 71% of staff in Hong Kong reported fatigue [13]. Prospective studies further indicated that fatigue intensified over time under pandemic or epidemic conditions [14,15]. Consistent risk factors reported in cross-sectional studies included long working hours, night shifts, heavy workloads and prolonged use of personal protective equipment (PPE) [16–20], as well as frontline working [21–24].

In addition to these occupational demands, the psychological burden of pandemic working has been significant. Many HCW experienced distress, anxiety, depressive and post-traumatic stress symptoms [25–31], all of which were strongly associated with fatigue. Among emergency mobile teams in Japan, for example, post-traumatic stress symptoms were associated with fatigue in a cross-sectional study of 331 HCW [32] and higher exhaustion correlated with higher anxiety among doctors and nurses working during COVID-19 [33,34]. Conversely, resilience, self-efficacy, - and adequate recovery between shifts [35–37] were inversely related to fatigue in cross-sectional studies, clearly demonstrating a role for certain protective factors.

Previous studies have indicated that HCW experience significant fatigue. Despite its clear implications for workforce wellbeing and performance [38], few reviews have comprehensively synthesized fatigue outcomes across multiple coronavirus public health emergencies. This systematic review and meta-analysis address this gap by quantifying the prevalence of fatigue among HCW during SARS, Middle East respiratory syndrome (MERS), and COVID-19 and identifying the associated risk and protective factors. Factors that affect multiple areas of work quality, motivation and satisfaction may be amenable to change through policy and practise aimed at mitigating fatigue, including resilience building. Therefore, this study examined the magnitude of the problem and level of risk with the aim of informing the development of healthcare strategies that support HCW in providing safe and effective healthcare during a public health emergency.

The objectives of this review were: (i) to examine the prevalence of fatigue among HCW working in a healthcare location during SARS, MERS or COVID-19, (ii) to investigate potential predictors associated with fatigue outcomes (e.g. depression, anxiety, post-traumatic stress disorder (PTSD), job role, shifts, etc.) and (iii) to synthesize the collected available data by conducting a meta-analysis.

## METHODS

The study protocol followed the PRISMA guidelines (Preferred Reporting Items for Systematic Reviews and Meta-Analyses) [39] and is available in Supplementary Document S1 (available as Supplementary data at *Occupational Medicine* Online). Prospero Registration No. CRD42021259045. PsycINFO, Ovid-Medline, CINAHL, Embase, HMIC and Cochrane Library were searched from the respective inception dates (Embase <1947; APA PsycINFO <1806; HMIC <1979; CINAHL 1984; Cochrane Library 1993) until 5 July 2024. The Boolean search string (multi-purpose) was: (SARS OR “severe acute respiratory syndrome” OR SARS-CoV-2) OR (coronavirus OR COVID-19 OR Covid* OR Covid19) OR (MERS OR “Middle East respiratory syndrome”) AND (fatigue* OR tired* OR exhaustion OR exhausted* OR lethargy*) AND (healthcare adj2 worker* OR healthcare adj2 professional*) OR HCW* OR (doctor OR physician* OR clinician* OR surgeon* OR nurse OR midwife OR midwives OR paramedic OR EMT OR general practitioner OR GP) OR (medical adj2 staff OR healthcare adj2 employee OR healthcare adj2 personnel OR healthcare adj2 provider). Database-specific modifications are available in Supplementary Document S2 (available as Supplementary data at *Occupational Medicine* Online). Reference lists of the included studies were also searched for additional eligible articles. Inclusion criteria were (i) primary articles in English; (ii) fatigue measured using valid fatigue scales (e.g. Chalder Fatigue Questionnaire), fatigue item(s) from ADQ (author designed questionnaires) (e.g. ‘did you feel exhausted or tired due to your work with COVID19 patients?’) or fatigue subscales (e.g. The Occupational Fatigue Exhaustion/Recovery (OFER) Scale); (iii) HCW including doctors, nurses, ancillary staff, healthcare and laboratory technicians, porters, administration, paramedics, physical, psychological or occupational therapists, and other staff working in any healthcare setting; (iv) data collected during MERS, SARS or COVID-19; (v) any study design (cohort, case-control, cross-sectional, randomized controlled trial); and (vi) only studies with quantitative fatigue data were included. Exclusion criteria were (i) compassion fatigue, decision fatigue, emotional exhaustion, emotional fatigue, moral fatigue or pandemic fatigue (defined as ‘disengagement with recommended protective behaviours, or seeking relevant information, general complacency and detachment’); (ii) ‘burnout’; (iii) newspaper articles, conference papers, abstracts, editorials, opinions or commentaries; and (iv) protocols, qualitative designs, systematic reviews/meta-analyses, case reports, vaccination or ‘transmission’ studies. To separate ‘virus-related fatigue’ from ‘work-related fatigue’, articles including HCW infected with a named virus were omitted.

Screening and data abstraction were performed independently by two researchers (H.W., K.P.W.). Deduplicated articles were imported into Rayyan for full screening [40], with 11% of the final included studies independently checked by the senior researcher (T.C.). Discrepancies between reviewers were resolved via discussion and consensus. Extracted data from the included studies were entered into a spreadsheet comprising the citation, population, sample size, control group, country of origin, virus type, study period, study design, inclusion/exclusion criteria, objectives, outcome variables (e.g. fatigue, exhaustion), associated variables (e.g. PTSD, depression), scales used, results and power calculation (Y/N). Articles fully screened, but not meeting our inclusion criteria, were recorded with reasons for the exclusion.

Risk of bias was assessed using the National Institutes of Health Quality Assessment Tool for Observational Cohort and Cross-sectional designs [41]. Appraisers are required to assign a ‘yes’, ‘no’, ‘cannot determine’, ‘not reported’ or ‘not applicable’ to each item. For quasi-experimental designs, the JBI (Joanna Briggs Institute) Critical Appraisal Checklist for Quasi-experimental designs was used [42]. For RCT (randomized controlled) designs, the JBI Critical Appraisal Checklist for was employed. Each item on the scale requires a ‘yes, ‘no’, ‘unclear’ or ‘not applicable’ rating. Overall study quality was graded as follows: cross-sectional/cohort designs, graded as ‘good’, ‘fair’ or ‘poor’ (representing the lowest risk to highest risk of bias); and RCTs/quasi-experimental designs graded as ‘low’, ‘moderate’ and ‘high’ risk of bias.

Descriptive statistics, including the frequency distributions of key variables and pooled statistics for the study sample, were conducted. Random effects models were used for all analyses. We computed a meta-analysis to estimate the pooled prevalence of fatigue with 95% confidence intervals for 32 studies. To reduce variability in the fatigue outcomes, only ‘chronic’ data were included in analyses where both ‘acute’ and ‘chronic’ fatigue data were measured by the OFER Scale. To maintain the independence of observations, only physical fatigue was entered into the overall analysis. Mental fatigue was calculated as an independent outcome. Studies using the terms ‘exhaustion’, ‘fatigue’ and ‘chronic fatigue’ were used interchangeably for ‘fatigue’ and entered all analyses. One study contained fatigue data during MERS and COVID-19 [43]. Therefore, only COVID-19 data were used in fatigue prevalence. If studies incorporated healthcare and non-healthcare professionals (e.g. community workers,) in the same study, only the HCW data were included. We examined the difference in the proportion of fatigue in doctors (i.e. surgeons, physicians, GPs) and nurses (including nurse technicians and nursing assistants) and between ‘frontline workers’ (defined as doctors, nurses, janitors, emergency medical technicians (EMT0, occupational or cognitive behavioural therapists, physiotherapists, phlebotomists, dentists, etc.)) and ‘non-frontline workers’ (defined as staff who were primarily not in contact with infected patients such as administration/office staff, medical technicians, auxiliary staff). Studies with no clear description of ‘frontline’ or ‘non-frontline’ were excluded from this analysis. Fatigue related to PPE was investigated as a discrete outcome. Sensitivity analyses were conducted on the main outcome to exclude the SARS studies and examine the difference in fatigue between studies, using a valid fatigue scale compared to non-validated fatigue measures (i.e. ADQ). Insufficient data for those who were not fatigued and a lack of control/comparison groups precluded additional meta-regressions or other analyses. Meta-analyses were conducted using R Studio, Version 4.3.1, using ‘metafor’, ‘meta’, ‘metasens’ and ‘ggplot’ [44]. Heterogeneity was assessed using Cochran *Q* statistic. We obtained the *I*^2^ statistic with the degree of heterogeneity categorized as ‘not important’ (0–40%), ‘moderate’ (30–60%), ‘substantial’ (50–90%) and ‘considerable’ (75–100%) [45]. Egger’s tests were used to explore potential publication bias for the main analysis and sub-groups with >10 studies.

This systematic review and meta-analysis used existing published data. Therefore, no ethical approval was sought during this research.

## RESULTS

**Figure 1.**
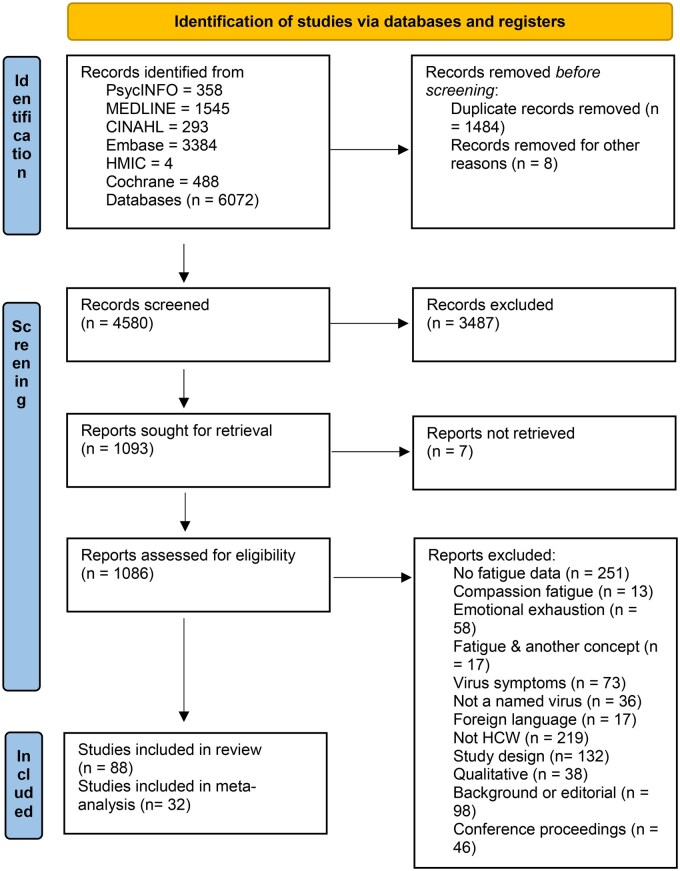
PRISMA flow chart.

Searches were conducted on the included databases from inception dates to 5 July 2024. A total of 6072 articles were identified from database searches. Following deduplication, the titles and abstracts of 4580 studies were screened against our inclusion criteria to assess suitability, resulting in 1093 eligible for full screening. A total of 88 articles met our inclusion criteria and were included in the review, and of these, 32 were eligible for a quantitative analysis. A PRISMA Flow Diagram is available in Figure 1.

A total of 74 914 participants from 88 studies were included. A complete summary of all 88 included articles is presented in Table S3 (available as Supplementary data at *Occupational Medicine* Online). Frontline staff comprised 50 978 (68%) of the participants, and non-frontline staff were 12 045 (16%). Nurses comprised 38 346 (51%) of the participants; 18 187 (24%) were doctors, and the remaining participants were pharmacists, physical or occupational therapists, laboratory or other medical technicians or HCW unspecified. The pooled mean age across all 88 studies was 35.9 years (SD = 8.3). Male participants made up 20%, and females made up 76% of the total sample. Table 1 indicates the key characteristics of all studies and the studies included for the meta-analysis.

**Table 1.** Pooled characteristics of all studies (*n* = 88) and studies included in the meta-analysis (*n* = 32)^a^

		All included studies (*n* = 88)		Fatigue studies included meta-analysis				Meta-analysis (*n* = 32)
	n		*n*	Reliable scales *n* (%)	*n*	Author-designed questionnaires *n* (%)	*n*	All fatigue studies *n* (%)
Gender distribution, Pooled *n* (%)								
Male	76	14 901 (20)	12	1282 (19)	16	5532 (81)	28	6814 (21)
Female	78	56 656 (76)	13	11 746 (13)	16	13 174 (41)	29	24 920 (77)
Other	3	423 (0.5)	1	416 (3)	–	–	1	416 (1)
Mean age, Pooled (*M* ± SD)	40	35.9 ± 8.1	8	35.9 ± 10.4	9	36.9 ± 10.5	17	38 ± 9.0
Frontline healthcare workers, Pooled *n* (%)								
Frontline	71	50 978 (81)	12	12 224 (56)	15	9583 (44)	27	21 807 (42)
Non-Frontline	62	12 045 (19)		872 (28)		3032 (98)	26	3904 (32)
Healthcare role, Pooled *n* (%)								
Nurses, Pooled *n* (%	63	38 346 (68)	10	7787 (43)	12	10 289 (57)	22	18 076 (32)
Doctors, Pooled *n* (%)	46	18 187 (32)	5	4452 (44)	14	5723 (56)	18	10 175 (26)

a The table summarizes the pooled proportions and means for key demographic and professional characteristics across all included studies (*n* = 88) and those included in the analyses (*n* = 32). Further distinctions are made between studies that used reliable, standardized scales (*n* = 13) and those that employed author-designed questionnaires (*n* = 19). Gender is represented as pooled proportions of male, female, and other participants, while age is reported as the pooled mean (*M*) and standard deviation (SD). Frontline status indicates the proportion of participants who were frontline healthcare workers versus non-frontline.

Countries or locations of origin were China, with 26 (29%) studies. Turkey, India and USA with seven studies each, and South Korea with five (6%). Italy had four (5%) studies and UK had three (3%). Greece, Malaysia, Japan, Germany and Brazil each had two studies. The remaining countries, Australia, Belgium, Canada, Ecuador, France, Jordan, Iran, Israel, Lithuania, Netherlands, Norway, Saudi Arabia, Serbia, Singapore and Vietnam had one study each. The remaining four studies involved participants from multiple countries. The most common context was COVID-19 (*n* = 85), and two studies related to SARS. One of the included studies investigated fatigue in the context of MERS and COVID-19 [46]. With respect to the year of publication, most COVID-19 studies (*n* = 25) were published during 2022. The next most frequent publication year was 2021, with 23 studies, followed by 18 studies in 2023 with and 14 studies in 2020; 2024 had six studies, and the remaining two SARS studies were published in 2004 and 2007.

Fifty studies (57%) used a valid fatigue measurement tool, 38 (43%) used an ADQ or questionnaire containing at least one item related to fatigue. The most utilized fatigue scale was the 14-item Fatigue Scale, employed by six studies, with the next most common being the Fatigue Severity Scale (FSS) and OFER scale used by five studies each. There were five RCTs, four quasi-experimental designs, two cohort designs, five prospective cohort study designs, and the remaining 72 were cross-sectional. Quality assessments were graded as ‘poor’, considered to have a high risk of bias (*n* = 17, 19%), ‘fair’ (*n* = 63, 71%) with a moderate risk of bias or ‘good’ (*n* = 8, 9%) with a low risk of bias. The quality of the evidence was considered low quality due to multiple methodological issues. Lower grades, for example, were allocated for a lack of control groups, selection bias, missing data, small sample size, or other methodological bias (lack of a validated and reliable scale), and non-randomization or ‘partial’ randomization not fully explained. Only 27 studies comprising the cohort and cross-sectional designs had >50% participation rates, and only one RCT was fully blinded to the treatment allocation. Most studies utilized self-selection methods and convenience, potentially affecting the reliability of the outcomes. Full quality assessment grades are available in Table S4 (available as Supplementary data at *Occupational Medicine* Online).

**Figure 2.**
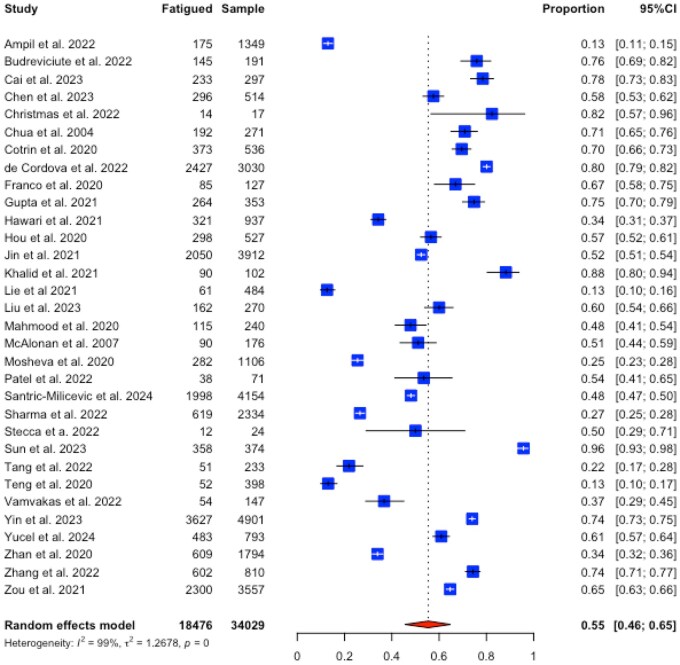
Forest plot of the proportion of fatigue in healthcare workers.

Thirty-two studies were included for a random-effects proportional analysis. A pooled prevalence of 55% (95% CI 46–65%, *I*^2^ 99%) was found among 34 029 participants (Figure 2). An Egger’s test was conducted to assess possible publication bias for the overall proportional analysis. Results were *t* = −0.41, *P* = 0.685 suggesting an absence of funnel plot asymmetry (Figure S5, available as Supplementary data at *Occupational Medicine* Online).

**Figure 3.**
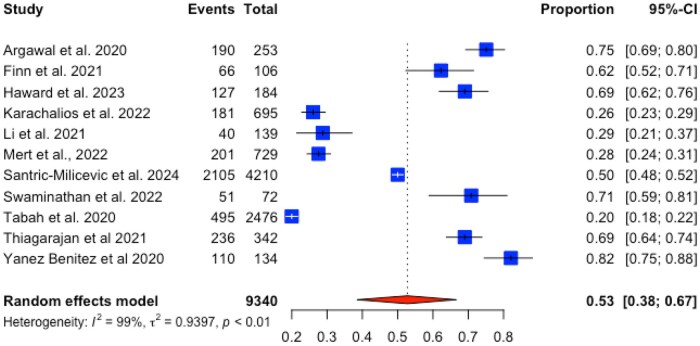
Forest plot of fatigue related to wearing PPE.

Four studies (3182 participants) had data related to ‘mental fatigue’; over half (58%, 95% CI 17–90%, *I*^2^ 99%) of the participants were fatigued. The sole association related to fatigue with sufficient data was that connected to wearing PPE. We computed a random effects meta-analysis pooling 11 studies, which found that 53% (95% CI 38–67%, *I*^2^ 99%) of HCW reported fatigue due to wearing items of PPE. A forest plot is available in Figure 3. An Egger’s test revealed no funnel plot asymmetry (*t* = 0.69, *P* = 0.509). The funnel plot is accessible in Figure S5 (available as Supplementary data at *Occupational Medicine* Online).

There were no differences in fatigue between doctors and nurses, and frontline compared to non-frontline staff. The proportion of doctors (*n* = 7813, 37%) with fatigue was 57% (95% CI 46–68%, *I*^2^ 98%, *k* = 11) and the proportion of nurses (*n* = 13 128, 63%) with fatigue was 67% (95% CI 52–79%, *I*^2^ 99.3%, *k* = 12), *P* = 0.327. An Egger’s test revealed no publication bias for either analysis (*P ≥* 0.05). The forest plot of this analysis is available in Figure S6 (available as Supplementary data at *Occupational Medicine* Online). No significant difference in fatigue was found between 4855 (19%) non-frontline staff (43%, 95% CI 32–55, *k* = 4) and 21 318 (81%) frontline staff (58%, 95% CI 45–69%, *k* = 21), *I*^2^ 99%, *P* = 0.103. Thirty studies investigated HCW fatigue during COVID-19, and two during SARS. Therefore, we conducted a sensitivity analysis excluding the SARS studies comprising 447 participants [13,22]. This resulted in an unchanged fatigue prevalence of 55% (95% CI 45–65%, *I*^2^ 99%). We compared the proportion of fatigue in each context and found no statistical difference between HCW employed during COVID-19 and those who worked during SARS (62%, 95% CI 47–74, *I*^2^ 94%, *k* = 2), *P* = 0.456.

**Figure 4.**
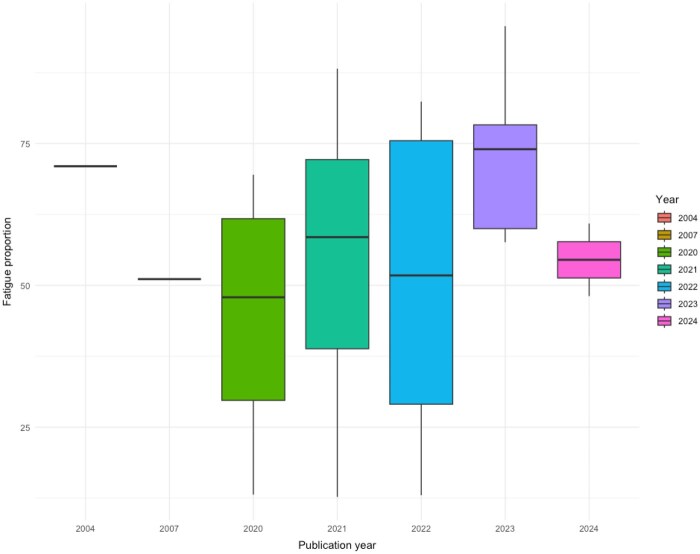
Box plot of fatigue prevalence by year of publication.

To explore sources of heterogeneity related to methodological aspects of the included studies (*n* = 32), we conducted a series of sensitivity analyses. Firstly, we examined the difference in the prevalence of fatigue between studies describing ‘physical exhaustion’ (50%, 95% CI 28–73, *k* = 7), ‘fatigue’ (58%, 95% CI 47–68, *k* = 22) and ‘tired’(48%, 95% CI 17–80, *k* = 3), which was not significant (*P* = 0.776). Secondly, we excluded seven studies rated as ‘high risk of bias’, which resulted in a marginal decrease in fatigue to 52% (95% CI 42–62%, *I*^2^ 99%, *k* = 25). A two-sided test for equality of proportions between fatigue in all studies (*n* = 32) and those high-risk studies removed (*n* = 25) was significant, *X*^2^(1) = 9.04, *P* = 0.002. There was no difference in fatigue between studies rated as ‘high’, ‘low’ or ‘moderate’ risk of bias (*P* = 0.474). The difference in fatigue between studies using a scale and those using an ADQ was not significant, *P* = 0.748. The proportion of fatigue in studies using a valid measurement tool (*n* = 13) was 57% (95% CI 41–72%, *I*^2^ 99%) compared to 54% (95% CI 42–66%) in 19 studies using an ADQ. Heterogeneity remained ‘substantial’, for all analyses suggesting that the variability of our results was not due to study quality, virus type or measurement tool. Finally, we analysed fatigue prevalence by year of publication (Figure 4), which revealed a significant difference in the proportion of fatigue between publication years: *Q*(6) = 28.7, *P* = 0.001, *k* = 32. The prevalence of fatigue reported in publication year 2023 (77%, 95% CI 59–89, *k* = 5) was significantly higher than other years.

Data on fatigue outcomes were available in 32 studies and, of these, only one association (PPE) had sufficient data to enable a quantified analysis. For other risk factors, the diversity of outcome measurements, scales and the small number of studies investigating each variable precluded a quantified analysis. Therefore, we illustrate the key variables and the direction of the correlations in a Table 2 for all 88 studies. A positive symbol (+) indicates a positive association, a negative symbol (−) indicates a negative association, and a zero (0) indicates no significant association between the investigated variable and fatigue [47]. The arrows indicate the direction of the association. For example, lower negative coping was associated with higher fatigue. The superscripts refer to the study reporting the association and correspond with the references.

**Table 2.** Key variables associated with fatigue^a^

Factor	Bivariate	Multivariate
Age	+^57^, 0^58^, +^36^, 0^59^, +^60^, 0^56^, +^47^, 0^23^, 0^35^, 0^48^, 0^64^, 0^54^, 0^49^, 0^26^	+^55^, 0^90^, –^62^, +^24^, 0^20^, +^61^, +^50^
Gender (F)	0^57^, 0^58^, 0^51^, 0∼^36^, 0^59^, 0^60^, 0^56^, +^47^, +^23^, +^35^, 0^62^, +^48^, +^54^, 0^26^	+^49^, +^20^, +^50^, +^52^, 0^61^, +^55^
Psychological		
Anxiety symptoms ↑	+^56^, 0^67^, +^34^, +^33^, +∼^26^, +^52^	+^62^, +∼^51^, +∼^25^, +^54^, +^27^, 0∼^26^, +^35^, +^21^, +^66^, +^65^, +^3^
Depressive symptoms ↑	+^34^, +^33^, +^52^	+^62^, +^59^, +^54^, +^66^, +^27^, +∼^26^, +^68^, +^21^, +^65^
Post-traumatic stress symptoms ↑	+^28^, +^36^	+^32^
Stress/Distress ↑	+^24^, +^34^, +^47^, +^35^, +^52^	+^62^, +^69^, 0^70^
Work-related		
Burnout	+^60^	+^76^, +^24^, +^12^
Infected patients contact/care	+^1^, +^50^, +^24^, +^47^, +^74^, +^59^	+^76^, 0^59^, +^23^, 0^90^, +^48^
Hours ↑	0^58^, +^75^, +^56^, +^47^, +^74^	+^20^, +^65^, 0^64^
Professional title (junior, senior)	0^49^, 0^36^, +* ^60^, +^26^, 0^35^, 0^56^	–* ^59^, 0^26^
Inter-shift recovery ↑	–^52^, –^47^	
Rest breaks↓	+^74^, +^86^	
Shifts (Day/night)	+^16^, +^47^, 0^74^	+^55^, 0^49^
Workload ↑	+^71^, +^72^, 0^52^	+^68^, +^23^
Years of experience/residency ↑	–^16^, 0^74^, 0^35^, +^47^, 0^36^, 0^59^, 0^62^	+^49^, 0^59^
Hesitation to work↑		+^10^, –∼^10^
Working environment (ventilation, safety etc)	+^118^, 0^67^, 0^97^	+^90^
Organizational support↑	–^88^	0^90^, –^65^
Other		
Fear of infection	+^58^, 0^71^, +^24^, +^56^, 0^97^	
Negative coping ↑	+^36^, +^28^	
Resilience ↑	–^35^, –^94^, –^88^	
Strength/optimism/tenacity	–^94^	0^90^
Self-efficacy ↑	–^36^	
Sleep duration/Sleep quality ↓	0^98^, +^57^, 0^49^, +^102^, +^99^, +^27^, +^88^, +^56^	+^62^, +^97^, 0^26^, +^101^, +^65^, +^100^

a Key: 0 no association; + positive association; − negative association; ↑ higher; ↓ lower, #between low/high Pittsburgh Sleep Quality Index scores & severe/mild fatigue; ∼with mental fatigue;

* junior HCW. Superscript numbers refer to the study reporting the association and correspond with the reference list.

Gender was reported in 20 studies with a trend for fatigue being higher in women [20,23,35,36,46,48–55]. Six studies found no association between gender and fatigue [56–61]. Overall, most studies found high mean fatigue in both genders. For instance, ‘chronic fatigue’ mean scores on the OFER were reported as 69.1 (± 27.8) for men and 72.9 (± 26.2) for women, *P* = 0.152 [58], a pattern found in other studies [36,50,62,63]. Reported effect sizes were sizeable. For example, a study of 4509 HCW found that both men (OR, 5.15) and women (OR, 6.30) had high levels of fatigue [61]. The majority were rated as low to moderate quality, but all were cross-sectional, limiting causality inferences.

Age was investigated by 21 cross-sectional studies with eight reporting an association [24,36,46,50,57,60–62], with inconsistent results. One study of 1414 nurses, found that 19- to 25-year-olds had higher fatigue scores (*M* = 68.3 ± 24.1) compared to the 26–35 and 36–58 age groups (*P* = 0.01) [46]. Other studies found that older age groups were significantly more tired [50,57,61]. HCW over 50 had slightly lower odds for fatigue (OR = 5.44) compared to <30 (OR = 5.70, *P* ≤ 0.05) [61], and lower mean fatigue scores (6.49 ± 4.09) than ≤29 years old (7.06 ± 4.63) on the FSS [36]. Many studies did not find a significant association between age groups and fatigue [20,23,35,48,56,59,64]. While these studies employed valid scales to measure fatigue improving their reliability, most had modest sample sizes and were cross-sectional designs with moderate quality assessments limiting generalizability.

Depressive symptoms were consistently associated with fatigue, with several studies reporting moderate to strong relationships [27,54,62,65–67]. Multivariate results [21,26,59,68] found higher fatigue risk associated with more severe depressive symptoms, ranging between OR = 1.69 (95% CI 1.48–1.94) and OR = 7.40 (95% CI 6.29–8.70). However, only two used a validated fatigue measure, limiting reliability. Correlational evidence showed stronger associations with chronic fatigue than acute fatigue: for instance, depressive symptoms were correlated with ‘acute’ fatigue at *r* = 0.374 and ‘chronic’ fatigue at *r *= 0.613, among frontline doctors [34], with similar results in a multi-regional study comprising 793 HCW: (‘chronic’ fatigue *r* = 0.522, and ‘acute’ fatigue *r* = 0.373, both *P* ≤ 0.01) [52].

Anxiety was examined in 16 studies, with most finding ­positive, strong associations with higher fatigue [21,27,33–35,52,54,56,62,65], and of note, with mental fatigue in two studies [25,51]. The two exceptions to this trend included a study of 233 [26] doctors, nurses and technicians, with authors reporting a positive association at the univariable level, but not at multivariable (OR = 1.01, *P* = 0.968). The other study found no correlation between state-trait anxiety and fatigue scores, *P* = 0.834 [67].

We also found that stress and distress had significant associations with fatigue [24,34,35,46,52,62,69,70]. For example, Mohd Fauzi *et al.* (2020) reported that stress scores correlated with chronic fatigue, *r* = 0.583, and acute fatigue, *r* = 0.405, all *P* ≤ 0.001. Another study investigating distress in 937 healthcare professionals found 32% experienced ‘high distress’ and this was associated with higher fatigue: OR = 2.40, *P* ≤ 0.001 [69]. All studies were cross-sectional, and most studies examining psychological correlates used valid but different fatigue instruments, making comparisons difficult. Further, it is possible that study samples included those with existing comorbid anxiety, depression or PTSD, making causal relationships difficult to determine.

Some work-related factors such as workload and working hours were associated with fatigue, with consistent results [20,23,46,52,56,58,64,71–74]. For instance, HCW who worked longer hours, particularly >12 h per day, and reported a higher workload were more fatigued [65,68]. Shift work was a consistent predictor of fatigue, with night shifts identified as one of the risk factors [16,46,55]. and longer shifts in another study, *P* = 0.001 [75]. Taken together, these factors indicate the high physical and mental work demands experienced by HCW, giving rise to fatigue [34]. Severe burnout was a work factor found to be related to higher fatigue in four studies [12,24,60,76]. Burnout risk, for example, was reported as 3.94 times higher (95% CI 2.80–5.56; *P* ≤ 0.001) among HCW with chronic fatigue symptoms [76]. Similarly, a strong relationship with burnout was found in two studies of nurses (*P* ≤ 0.001) [12,24].

Other noteworthy relationships included exposure to infected patients which was found to be significantly related to higher fatigue in most studies investigating this factor [1,24,46,50,59,74,76]. For instance, in a large study of over 7000 HCW, van Roekel *et al.* (2021) found that direct contact with COVID-19 patients was significantly related to higher physical exhaustion (*d *= 0.223, *P* ≤ 0.01). Interestingly, chronic fatigue was correlated with cumulative days of care for infected patients (OR = 1.18; 95% CI 1.02-1.37), suggestive of the toll of caring for sick patients [76]. Years of experience and seniority were inconsistently related to fatigue, with five studies reporting no association [26,35,36,62,74] and four studies [16,46,49,59] finding a relationship with fatigue. However, the trend was not clear. For example, Jin *et al.* (2021) found that frontline junior clinicians had lower odds for fatigue: OR = 0.82, *P* = 0.045, while ≥15 years of work experience was related to fatigue in multivariable analysis of frontline nurses: β = 0.09, *P* = 0.013 [59]. Ortabag *et al.* (2022) found that nurses with 2–4 years’ experience reported higher fatigue scores than both less experienced and more experienced nurses, *P* = 0.001. All studies examining work-related factors were cross-sectional, including 10 that utilized author-designed questionnaires. Further, most of the associations were weak to modest in strength.

## DISCUSSION

This systematic review and meta-analysis of 88 studies found that over half of healthcare professionals experienced fatigue while working during COVID-19 or SARS (55%, 95% CI 46–65%) and that a significant proportion reported mental fatigue (58%). Our results correspond with one other pre-print review of 48 studies [77] reporting a chronic fatigue prevalence of 51% (95% CI 47–55%). Other reviews have synthesized data in narrative form [5] or have not been in the context of a coronavirus [78,79]. Nevertheless, our results indicate that rates of fatigue were higher compared to pre-pandemic levels, 42% in one large study [11].

The majority of participants in this review were frontline nurses and females, which accords with previous systematic reviews. Nurses, for instance, were consistently found to have higher fatigue [5,80], as were those working on the frontline with infected patients [81], although we did not find a significant statistical difference between frontline and non-frontline staff. Fear of infection, as reported in several studies [56], may partially explain this, as concerns about becoming ill and transmitting infection to family members, added to the high psychological burden [3,22] Although we found that 53% of HCW were fatigued due to wearing PPE, a quasi-experimental study did not find concomitant reductions in cardiopulmonary resuscitation (CPR) performance [82]. However, extended or prolonged PPE use may be a more important risk factor for fatigue [83–86]. Confidence in infection control and PPE training also appeared protective, reducing fatigue and stress during SARS [13] and COVID-19 [9,87]. Taken together, the perception of having sufficient protection, alongside organizational support [65,88,89] had the effect of mitigating fatigue [52]. This highlights the importance of equipping frontline workers and ensuring competencies in equipment handling/use in limiting fatigue [37].

We also found strong associations between fatigue and psychological outcomes, including depressive symptoms [62,65,66] anxiety [25,34,51], post-traumatic stress symptoms [28,32] and stress/distress [35,69]. Our findings suggest a relationship between tiredness and psychological sequelae, significant impact on well-being and the need for fatigue management alongside psychological screening and support [90,91]. Persistent fatigue may lead to low mood and depressive symptoms. Equally, significant stressors such as anxiety about transmitting the virus or becoming infected [92] may have led to fatigue. However, Mohd Fauzi *et al.* (2020) found that the prevalence of symptoms of depression, anxiety and stress among doctors were similar to population norms. Similarly, Chua *et al.* (2004) found that HCW were no more ‘stressed’ than matched healthy controls during the SARS epidemic. This may signal the importance of individual factors, such as resilience. Resilience is described as ‘the ability to withstand significant change, adversity or challenge’, accompanied by a number of traits such as optimism, resourcefulness and hardiness [93]. In our review, resilience was inversely related to fatigue [35,88,94], strength, optimism and tenacity [94] and self-efficacy [36]. When resilience is higher, it is possible that more energy is available to deal with adverse workloads and other job-demands implicated in fatigue. More resilient nurses, for instance, tended to have more energy and an ability to regulate their emotional states [37].

Organizational factors such as working hours, shift length/type and contact with infected patients are acknowledged to be problematic for fatigue in healthcare [78,95]. Our results support this evidence: long hours and increased workload were consistently related to higher fatigue across professions [52,56,68,71,75]. These demands were associated with fewer breaks and less sleep, depleting resources and increasing fatigue [96]. Several studies in this review found that insomnia/sleep [62,97–102], taking shorter, fewer or no breaks [74] and lower inter-shift recovery (the rest period between shifts) were related to higher fatigue [46,52], consistent with previous research [79]. Thus, fatigue is driven, in part, by a combination of high demands and insufficient recovery opportunities. The Job Demands-Resources model [103] provides a framework for interpreting these findings. The model proposes that employee wellbeing depends upon the interaction between job demands such as workload, shift patterns or emotional distress, and job resources including organizational support, rest and personal resilience. When demands outweigh resources, strain and fatigue may develop. Evidence from our review reflects this imbalance during COVID-19: high demands such as long hours, heavy caseloads and extended PPE use were linked to increased fatigue [20,46,71,83,104], while resources such as adequate breaks, resilience and organizational support lessened its impact [35,75,88]. This interpretation suggests that interventions should extend beyond fostering individual coping skills to include organizational strategies that lower excessive demands and increase resources. These include ensuring sufficient staffing, limited shift lengths and enabling sufficient rest/recovery, which may relieve fatigue [105–107]. Few interventions are designed for pandemic conditions, most were developed and tested under ‘normal’ conditions. Although there is limited evidence for the long-lasting effects of resilience training [108], evidence supports resilience training for fatigue [109]. Furthermore, organizational policies such as a supportive workplace culture, increased autonomy in decision-making and leadership reduce fatigue levels. Additionally, a ‘fatigue risk management plan’ should be in place [110,111].

Our findings are limited by several factors. Firstly, the pooled estimates were highly heterogeneous (∼99%), not fully explained by methodological differences such as measurement tools, indicating caution in interpreting the results. Another key limitation was the reliance on cross-sectional designs in the meta-analysis and narrative review. We were, therefore, unable to disentangle whether working hours directly lead to fatigue, for example, or if symptoms of anxiety/depression contributed to the onset of fatigue. The lack of longitudinal data limited our ability to track changes in fatigue across different stages or in response to organizational changes. Therefore, our results are limited about the direction of causality and should be interpreted with caution. Additionally, our prevalence (*n* = 32) was affected by the high risk of biased studies, possibly inflating the proportion of HCW with fatigue. The lack of pre-pandemic or epidemic fatigue data contributed to the overall low confidence in our results. Moreover, the broad range of measures employed and diverse definitions of fatigue (e.g. fatigue, tiredness), with the use of unvalidated questionnaires with differing fatigue cut-offs, made comparisons across studies problematic. Future epidemiological research should employ prospective designs to examine fatigue trends and employ more rigorous methods for measuring fatigue. Although we excluded studies that included HCW who were infected with a virus, we acknowledge that virus-related fatigue symptoms may have contributed to our results. Different equipment types, for example, N95 or surgical masks, listed in the studies were included for the PPE-related fatigue, meaning our results could be a result of such differences and may explain the variance. However, all studies listed commonalities including gloves, goggles/visors, gowns and masks [112] providing enough similarities for confidence in these results.

The majority of the intervention studies [113–115] focused on reducing fatigue using individual-level approaches such as workout [116] or wellness [117] programmes, but future studies should investigate ergonomic and functional processes found to affect fatigue [118]. These may include ward layouts, noise levels, temperature and spatial privacy [119]. We were unable to quantify risk factors associated with fatigue due to methodological differences, which should be addressed in future studies. A large body of research has focused on the psychological effects of working during an epidemic/pandemic [120,121], leaving some gaps for future research such as fear of being infected and infecting others. These are common themes in most SARS or COVID-19 studies involving HCW but have received limited attention in relation to fatigue. Also, social support is a resource reducing the severity of stress/distress [122] and may positively aid fatigue, pointing to an area for future research.

In conclusion, this review found that some workplace practises may compromise wellbeing by contributing to fatigue outcomes, while structures such as organizational support and infection control may protect against worse fatigue. Although individual differences in resilience and self-efficacy were associated with lower fatigue, relying on these factors alone neglects the organizational changes required to address systematic drivers of fatigue. Mitigating such negative effects requires reducing job demands, such as extended shifts and heavy workloads, while strengthening resources including improved staffing levels, rest breaks, reduced hours and psychological support [121,123]. Our study highlights that fatigue is multi-dimensional, requiring coordinated interventions at both the individual and organizational levels to safeguard HCW health and sustain workforce resilience in future public health emergencies.

## Supplementary Material

kqag015_Supplementary_Data

## Data Availability

Data are available on request from the corresponding author.
