# Supplementary material for: Healthcare worker fatigue during COVID-19, SARS, and MERS: a meta-analysis
Source: Occup Med (Lond). 2026 Mar 7;76(2):132–43. doi: 10.1093/occmed/kqag015 (PMC13095218; doi:10.1093/occmed/kqag015)
Supplement: kqag015_Supplementary_Data [file kqag015_supplementary_data.zip › 25-OP-062.R2 Supplementary Table S4 Quality Assessment Grades FINAL.doc]

Supplementary S4 Quality assessments

Observational cohort and cross-sectional studies

| **Study** | ***Was the research question or objective in this paper clearly stated?*** | ***Was the study population clearly specified & defined?*** | ***Was the participation rate of eligible persons at least 50%?*** | ***Were all the subjects selected or recruited from the same/ similar populations & same time period? Were inclusion & exclusion criteria for being in the study prespecified & applied uniformly to all participants?*** | ***Was a sample size justification, power description, or variance & effect estimates provided?*** | ***For the analyses in this paper, were the exposure(s) of interest measured prior to the outcome(s) being measured?*** | ***Was the timeframe sufficient so that one could reasonably expect to see an association between exposure & outcome if it existed?*** | ***For exposures that can vary in amount or level, did the study examine different levels of the exposure as related to the outcome (e.g., categories of exposure, or exposure measured as continuous variable)?*** | ***Were the exposure measures (independent variables) clearly defined, valid, reliable, & implemented consistently across all study participants?*** | ***Was the exposure assessed more than once over time?*** | ***Were the outcome measures (dependent variables) clearly defined, valid, reliable, & implemented consistently across all study participants?*** | ***Were the outcome assessors blinded to the exposure status of participants?*** | ***Was loss to follow-up after baseline 20% or less?*** | ***Were key potential confounding variables measured & adjusted statistically for their impact on the relationship between exposure(s) & outcome(s)?*** | ***Rating*** |
| --- | --- | --- | --- | --- | --- | --- | --- | --- | --- | --- | --- | --- | --- | --- | --- |
| *Agarwal et al. 2020* | ✓ | ✓ | ✓ | ✓ | ✓ | ✗ | ✓ | ✓ | ✗ | ✗ | ✗ | ✗ | NA | ✗ | Fair |
| *Akinici & Basar 2021* | ✓ | ✓ | ? | ✓ | ✗ | ✗ | NA | ✓ | ✓ | NA | ✓ | ✗ | NA | ✗ | Fair |
| *Ampil et al. 2022* | ✓ | ? | ? | ? | ✗ | ✗ | NA | ✓ | ✗ | NA | ✗ | ✗ | ✗ | ✗ | Poor |
| *Asaoka et al. 2020* | ✓ | ✓ | ✗ | ✓ | ✗ | ✗ | ✓ | ✓ | ✗ | ✗ | ✓ | ✗ | NA | ✓ | Fair |
| *Barpanda et al. 2023* | ✓ | ✓ | ? | Y | Y | ✗ | ✗ | ✗ | ✓ | NA | ✓ | ✗ | NA | ✗ | Poor |
| *Budrfeviciute et al. 2022* | ✓ | ✓ | ✗ | ✓ | ✓ | ✗ | ✗ | ✓ | ✗ | NA | ✗ | ✗ | NA | ✗ | Poor |
| *Cai et al. 2023* | ✓ | ✓ | ? | ✓ | ✗ | ✗ | ✓ | ✓ | ✗ | ✗ | ✗ | ✗ | NA | ✗ | Fair |
| *Caliskan et al. 2022* | ✓ | ✓ | ? | ✓ | ✗ | ✗ | NA | ✓ | ✓ | NA | ✓ | ✗ | NA | ✗ | Fair |
| *Chen et al. 2023* | ✓ | ✓ | ? | ✓ | ✓ | ✗ | ✗ | ✓ | ✓ | ✗ | ✓ | ✗ | NA | ✓ | Fair |
| *Cho et al. 2023* | ✓ | ✓ | ✓ | ? | ✓ | ✗ | ✗ | ✓ | ✓ | NA | ✓ | ✗ | NA | ✓ | Fair |
| *Christmas et al. 2022* | ✓ | ✗ | ✓ | ? | ✗ | ✗ | ✗ | ✗ | ✓ | NA | ✗ | ✗ | NA | ✗ | Poor |
| *Costa et al. 2021* | ✓ | ✓ | ✓ | ✓ | ✗ | ✗ | ✗ | ✓ | ✓ | NA | ✓ | ✗ | NA | ✓ | Fair |
| *Cotrin et al. 2020* | ✓ | ✓ | ✓ | ✓ | ✓ | ✗ | ✗ | ✓ | ✗ | NA | ✗ | ✗ | NA | ✗ | Fair |
| *Dai et al. 2023* | ✓ | ✗ | ? | ? | ✗ | ✗ | ✗ | ✓ | ✓ | NA | ✗ | ✗ | NA | ✓ | Poor |
| *De Cordova et al. 2022* | ✓ | ✓ | ✗ | ✓ | ✗ | ✗ | ✗ | ✓ | ✗ | NA | ✗ | ✗ | NA | ✓ | Poor |
| *Duong-Quy et al. 2022* | ✓ | ✓ | ? | ? | ✗ | ✓ | ? | ✓ | ✓ | ✓ | ✓ | ✓ | ? | ✗ | Fair |
| *Engelmann et al. 2023* | ✓ | ✓ | ? | ✓ | ✗ | ✓ | ✓ | ✓ | ✓ | ✓ | ✓ | ✗ | ✗ | ✓ | Fair |
| *Erdagi Oral et al. 2022* | ✓ | ✗ | ✓ | ? | ✓ | ✗ | ✗ | ✓ | ✓ | NA | ✓ | ✗ | NA | ✗ | Poor |
| *Finn et al. 2021* | ✓ | ✓ | ✓ | ✓ | ✗ | ✗ | ✓ | ✓ | ✓ | ✗ | ✗ | ✗ | NA | ✗ | Fair |
| *Franco et al. 2020* | ✓ | ✓ | ✓ | ✓ | ✗ | ✗ | ✓ | ✓ | ✗ | ✗ | ✗ | ✗ | NA | ✗ | Fair |
| *Frenkel et al. 2022* | ✓ | ✓ | ? | ✓ | ✗ | ✓ | ✓ | ✓ | ✗ | ✓ | ✓ | ? | ✓ | ✓ | Fair |
| *Grelier et al. 2022* | ✓ | ✓ | ? | ✓ | ✗ | ✗ | ✗ | ✓ | ✓ | NA | ✗ | ✗ | NA | ✗ | Poor |
| *Gupta et al. 2021* | ✓ | ✓ | ? | ✓ | ✗ | ✗ | ✗ | ✓ | ✓ | NA | ✓ | ✗ | NA | ✓ | Fair |
| *Haward et al. 2023* | ✓ | ✓ | ? | ✓ | ✗ | ✗ | ✗ | ✓ | ✓ | NA | ✗ | ✗ | NA | ✗ | Poor |
| *Hawari et al. 2021* | ✓ | ✓ | ? | ✓ | ✗ | ✗ | ✓ | ✓ | ✓ | ✗ | ✓ | ✗ | NA | ✓ | Fair |
| *Hou et al. 2020* | ✓ | ✓ | ✓ | ✓ | ✓ | ✗ | ✓ | ✓ | ✓ | ✗ | ✓ | ✗ | NA | ✓ | Good |
| *Hou et al. 2022* | ✓ | ✓ | ? | ✓ | ✗ | ✗ | ✗ | ✓ | ✓ | NA | ✓ | ✗ | NA | ✓ | Fair |
| *Hwang et al. 2023* | ✓ | ✓ | ✓ | ✓ | ✓ | ✗ | ✗ | ✓ | ✓ | NA | ✓ | ✗ | NA | ✓ | Fair |
| *Jarrar et al. 2023* | ✓ | ✓ | ? | ✓ | ✓ | ✗ | ✗ | ✓ | ✓ | NA | ✓ | ✗ | NA | ✗ | Fair |
| *Jin et al. 2021* | ✓ | ✓ | ? | ✓ | ✗ | ✗ | ✗ | ✓ | ✓ | NA | ✓ | ✗ | NA | ✓ | Fair |
| *Karachalios et al. 2022* | ✓ | ✓ | ? | ✓ | ✗ | ✗ | ✗ | ✗ | ✓ | NA | ✗ | ✗ | NA | ✗ | Poor |
| *Khalid et al. 2021* | ✓ | ? | ✓ | ✓ | ✗ | ✗ | ✗ | ✓ | ✗ | NA | ✗ | ✗ | NA | ✗ | Poor |
| *Kishi et al. 2022* | ✓ | ✓ | ? | ✓ | ✗ | ✗ | ✗ | ✓ | ✓ | NA | ✓ | ✗ | NA | ✓ | Fair |
| *Li, Guo et al. 2022* | ✓ | ✓ | ? | ✓ | ✗ | ✗ | ✗ | ✓ | ✓ | NA | ✓ | ✗ | NA | ✓ | Fair |
| *Li, Jiang et al. 2021* | ✓ | ✓ | ✓ | ✓ | ✗ | ✗ | ✗ | ✓ | ✓ | NA | ✓ | ✗ | NA | ✓ | Fair |
| *Lie et al. 2021* | ✓ | ✓ | ? | ✓ | ✗ | ✗ | ✗ | ✓ | ✓ | NA | ✗ | ✗ | NA | ✓ | Fair |
| *Liu et al. 2023* | ✓ | ✓ | ? | ✓ | ✗ | ✗ | ✗ | ✓ | ✓ | NA | ✓ | ✗ | NA | ✓ | Fair |
| *Ma et al. 2021* | ✓ | ✓ | ? | ✓ | ✗ | ✗ | ✗ | ✓ | ✗ | NA | ✗ | ✗ | NA | ✗ | Poor |
| *Mahmood et al. 2020* | ✓ | ✓ | ? | ✓ | ✗ | ✗ | ✓ | ✓ | ✗ | ✗ | ✗ | ✗ | NA | ✓ | Fair |
| *Mannocci et al. 2023* | ✓ | ✓ | ✗ | ✓ |  | ✗ | ✗ | ✓ | ✓ | NA | ✗ | ✗ | NA | ✓ | Fair |
| *McAlonan et al. 2007* | ✓ | ✓ | ? | ✓ | ✗ | ✗ | ✓ | ✓ | ✓ | ✓ | ✓ | ? | ? | ✓ | Fair |
| *Mert et al. 2021* | ✓ | ✓ | ? | ✓ | ✗ | ✗ | ✗ | ✓ | ✓ | NA | ✓ | ✗ | NA | ✗ | Fair |
| *Mohd Fauzi et al. 2020* | ✓ | ✓ | ? | ✓ | ✓ | ✗ | ✓ | ✓ | ✓ | ✗ | ✓ | ✗ | NA | ✓ | Fair |
| *Mosheva et al. 2020* | ✓ | ✓ | ? | ✓ | ✗ | ✗ | ✓ | ✓ | ✓ | ✗ | ✓ | ✗ | NA | ✓ | Fair |
| *Nazario et al. 2023* | ✓ | ✓ | ✓ | ✓ | ✓ | ✗ | ✗ | ✓ | ✓ | NA | ✓ | ✗ | NA | ✓ | Fair |
| *Ortabag et al. 2022* | ✓ | ? | ✓ | ✓ | ✓ | ✗ | ✗ | ✓ | ✓ | NA | ✓ | ✗ | NA | ✗ | Fair |
| *Patel et al. 2022* | ✗ | ✓ | ✗ | ✓ | ✗ | ✓ | ✓ | ✓ | ✓ | ✓ | ✗ | ✗ | ✗ | ✗ | Fair |
| *Sagherian et al. 2023* | ✓ | ✓ | ✗ | ? | ✓ | ✗ | ✓ | ✓ | ✓ | ✗ | ✓ | ✗ | NA | ✗ | Fair |
| *Santric-Milicevic et al. 2024* | ✓ | ✓ | ✓ | ✓ | ✗ | ✗ | ✗ | ✓ | ✗ | NA | ✗ | ✗ | NA | ✗ | Fair |
| *Sharma et al. 2022* | ✓ | ✓ | ? | ✓ | ✗ | ✗ | ✗ | ✓ | ✓ | NA | ✓ | ✗ | NA | ✓ | Fair |
| *Sikaras et al. 2023* | ✓ | ✗ | ✓ | ✓ | ✓ | ✗ | ✗ | ✓ | ✓ | NA | ✓ | ✗ | NA | ✓ | Fair |
| *Song et al. 2023* | ✓ | ✓ | ? | ✓ | ✓ | ✗ | ✗ | ✓ | ✓ | NA | ✓ | ✗ | NA | ✓ | Fair |
| *Soylu et al. 2023* | ✓ | ✓ | ? | ✓ | ✗ | ✗ | ✗ | ✓ | ✓ | NA | ✓ | ✗ | NA | ✓ | Fair |
| *Stecca et al. 2022* | ✓ | ✓ | ? | ✓ | ✗ | ✗ | ✗ | ✗ | ✓ | NA | ✓ | ✗ | NA | ✗ | Poor |
| *Sun et al. 2023* | ✓ | ✓ | ? | ✓ | ✓ | ✗ | ✗ | ✓ | ✗ | NA | ✗ | ✓ | NA | ✓ | Fair |
| *Swaminathan et al. 2022* | ✓ | ✓ | ✗ | ✓ | ✗ | ✗ | ✓ | ✓ | ✗ | ✗ | ✓ | ✗ | NA | X | Fair |
| *Tabah et al. 2020* | ✓ | ✓ | ✓ | ✓ | ✗ | ✗ | ✓ | ✓ | ✗ | ✗ | ✗ | ✗ | NA | ✓ | Fair |
| *Tang et al. 2022* | ✓ | ✓ | ? | ✓ | ✗ | ✗ | ✗ | ✓ | ✓ | NA | ✓ | ✗ | NA | ✓ | Fair |
| *Teng et al. 2020* | ✓ | ✓ | ? | ✓ | ✓ | ✗ | ✓ | ✓ | ✓ | ✗ | ✓ | ✗ | NA | ✓ | Good |
| *Teo et al. 2021* | ✓ | ✓ | ✗ | ✓ | ✗ | ✗ | ✓ | ✓ | ✓ | ✗ | ✓ | ✗ | NA | ✓ | Fair |
| *Thiagarajan et al. 2021* | ✓ | ✓ | ? | ✓ | ✗ | ✗ | ✓ | ✓ | ✗ | ✗ | ✓ | ✗ | NA | ✓ | Fair |
| *Vamvakas et al. 2022* | ✓ | ✓ | ✓ | ✓ | ✓ | ✗ | ✗ | ✗ | ✗ | NA | ✓ | ✗ | ✗ | ✗ | Fair |
| *Vanhaecht et al. 2021* | ✓ | ✓ | ✗ | ✓ | ✗ | ✗ | ? | ✓ | ✗ | ✗ | ✗ | ✗ | NA | ✓ | Fair |
| *van Roekel et al. 2021* | ✓ | ✓ | ? | ✓ | ✗ | ✗ | ✓ | ✓ | ✗ | ✗ | ✗ | ✗ | NA | ✓ | Fair |
| *Wang et al. 2021* | ✓ | ✓ | ? | ✓ | ✗ | ✗ | ✓ | ✓ | ✓ | ✗ | ✓ | ✗ | NA | ✓ | Fair |
| *Wu et al. 2021* | ✓ | ✓ | ✓ | ✓ | ✗ | ✗ | ✗ | ✓ | ✓ | NA | ✓ | ✗ | NA | ✓ | Fair |
| *Xu et al. 2024* | ✓ | ✓ | ✓ | ✓ | ✓ | ✗ | ✗ | ✓ | ✓ | NA | ✓ | ✗ | NA | ✓ | Good |
| *Yanez Benitez et al. 2020* | ✓ | ✓ | ✗ | ✓ | ✗ | ✗ | ✓ | ? | ✗ | ✗ | ✗ | ✗ | NA | ✗ | Poor |
| *Yin et al. 2023* | ✓ | ✓ | ✓ | ✓ | ✓ | ✗ | ✗ | ✓ | ✓ | NA | ✓ | ✗ | NA | ✓ | Fair |
| *Yucel et al. 2024* | ✓ | ✓ | ✓ | ✓ | ✓ | ✗ | ✗ | ✓ | ✓ | NA | ✓ | ✗ | NA | ✓ | Good |
| *Zhan et al. 2020* | ✓ | ✓ | ✓ | ✓ | ✓ | ✗ | ✓ | ✓ | ✗ | ✗ | ✓ | ✗ | NA | ✓ | Fair |
| *Zhang, Hu et al. 2021* | ✓ | ✓ | ✓ | ✓ | ✗ | ✗ | ✗ | ✓ | ✓ | NA | ✓ | ✗ | NA | ✓ | Good |
| *Zhang, Liu et al. 2023* | ✓ | ✓ | ✓ | ✓ | ✗ | ✗ | ✗ | ✓ | ✓ | NA | ✓ | ✗ | NA | ✓ | Fair |
| *Zhang et al. 2022* | ✓ | ✓ | ? | ✓ | ✗ | ✗ | ✗ | ✓ | ✓ | NA | ✓ | ✗ | NA | ✓ | Fair |
| *Zhang, Xu et al. 2021* | ✓ | ✓ | ? | ✓ | ✗ | ✗ | ✗ | ✓ | ✓ | NA | ✓ | ✗ | NA | ✓ | Fair |
| *Zhou et al. 2021* | ✓ | ✓ | ✓ | ✓ | ✗ | ✓ | ✓ | ✓ | ✓ | ✓ | ✓ | ✗ | ✓ | ✓ | Good |
| *Zou et al. 2021* | ✓ | ✓ | ? | ✓ | ✓ | ✗ | ✓ | ✓ | ✓ | ✗ | ✓ | ✗ | NA | ✓ | Fair |

Key ✗= No; ✓= Yes; ? = Not clear; NA = Not applicable

**Case-control designs**

| **Study** | **1** | **2** | **3** | **4** | **5** | **6** | **7** | **8** | **9** | **10** | **11** | **12** | **Rating** |
| --- | --- | --- | --- | --- | --- | --- | --- | --- | --- | --- | --- | --- | --- |
| Chua et al. 2004 | ✓ | ✗ | ✗ | ✓ | ? | ✓ | ✓ | ? | ✓ | ✗ | ? | ✗ | Poor |
| Pearman et al. 2020 | ✓ | ✓ | ✗ | ✓ | ✓ | ✓ | ? | ✓ | ✗ | ✓ | ? | ✗ | Fair |

Key ✗= No; ✓= Yes; ? = Not clear; NA = Not applicable

NIH Quality assessment for case-control study: Questions

1. Was the research question or objective in this paper clearly stated and appropriate?
2. Was the study population clearly specified and defined?
3. Did the authors include a sample size justification?
4. Were controls selected or recruited from the same or similar population that gave rise to the cases (including the same timeframe)?
5. Were the definitions, inclusion and exclusion criteria, algorithms or processes used to identify or select cases and controls valid, reliable, and implemented consistently across all study participants?
6. Were the cases clearly defined and differentiated from controls?
7. If less than 100 percent of eligible cases and/or controls were selected for the study, were the cases and/or controls randomly selected from those eligible?
8. Was there use of concurrent controls?
9. Were the investigators able to confirm that the exposure/risk occurred prior to the development of the condition or event that defined a participant as a case?
10. Were the measures of exposure/risk clearly defined, valid, reliable, and implemented consistently (including the same time period) across all study participants?
11. Were the assessors of exposure/risk blinded to the case or control status of participants?
12. Were key potential confounding variables measured and adjusted statistically in the analyses? If matching was used, did the investigators account for matching during study analysis?

**Quasi experimental designs**

| **Study** | **Is it clear in the study what is the ‘cause’ and what is the ‘effect’ (i.e. there is no confusion about which variable comes first)?** | **Were the participants included in any comparisons similar?** | **Were the participants included in any comparisons receiving similar treatment/care, other than the exposure or intervention of interest?** | **Was there a control group?** | **Were there multiple measurements of the outcome both pre and post the intervention/exposure?** | **Was follow up complete and if not, were differences between groups in terms of their follow up adequately described and analyzed?** | **Were the outcomes of participants included in any comparisons measured in the same way?** | **Were outcomes measured in a reliable way?** | **Was appropriate statistical analysis used?** | **Rating** |
| --- | --- | --- | --- | --- | --- | --- | --- | --- | --- | --- |
| Baek e al. 2022 | ✓ | ✓ | ✓ | ✓ | ✓ | ✓ | ✓ | ✓ | ✓ | Low |
| Bodnar et al. 2024 | ✓ | ? | ✓ | ✗ | ✓ | ? | ✓ | ✓ | ✓ | Moderate |
| Giordano et al. 2020 | ✓ | ? | ✓ | ✗ | ✓ | ✗ | ✓ | ✓ | ✓ | Moderate |
| Serin & Calgar 2021 | ✓ | ? | ✓ | ✓ | ✗ | ✓ | ✓ | ✓ | ✓ | Moderate |

**Randomised controlled trials**

| **Study** | **Question Number** | | | | | | | | | | | | | **Rating** |
| --- | --- | --- | --- | --- | --- | --- | --- | --- | --- | --- | --- | --- | --- | --- |
|  | **1** | **2** | **3** | **4** | **5** | **6** | **7** | **8** | **9** | **10** | **11** | **12** | **13** |  |
| *Bektaş Akpınar et al. 2024* | ✓ | ✓ | ✓ | ✗ | ✗ | ✓ | ✓ | ✓ | ✓ | ✓ | ✓ | ✓ | ✓ | Moderate |
| *Ha et al. 2022* | ✓ | ✓ | ✓ | ✓ | ✗ | ✓ | ✓ | ✓ | ✓ | ✓ | ✓ | ✓ | ✓ | Moderate |
| *Karimi et al. 2024* | ✓ | ✓ | ✓ | ✓ | ✓ | ✓ | ✓ | ✓ | ✓ | ✓ | ✓ | ✓ | ✓ | Low |
| *Rauch et al. 2021* | ? | ✗ | ✓ | ✗ | ✗ | ? | ✓ | ✓ | ✓ | ✓ | ✓ | ✓ | ✓ | High |
| *Su et al. 2021* | ? | ✗ | ✓ | ✗ | ✗ | ? | ✓ | ✓ | ✓ | ✓ | ✓ | ✓ | ✗ | High |

Key ✗= No; ✓= Yes; ? = Not clear; NA = Not applicable

JBI Critical appraisal checklist for randomized controlled trials: Questions

1. Was true randomization used for assignment of participants to treatment groups?
2. Was allocation to treatment groups concealed?
3. Were treatment groups similar at the baseline?
4. Were participants blind to treatment assignment?
5. Were those delivering treatment blind to treatment assignment?
6. Were outcomes assessors blind to treatment assignment?
7. Were treatment groups treated identically other than the intervention of interest?
8. Was follow up complete and if not, were differences between groups in terms of their follow up adequately described and analyzed?
9. Were participants analyzed in the groups to which they were randomized?
10. Were outcomes measured in the same way for treatment groups?
11. Were outcomes measured in a reliable way?
12. Was appropriate statistical analysis used?
13. Was the trial design appropriate, and any deviations from the standard RCT design (individual randomization, parallel groups) accounted for in the conduct and analysis of the trial?
