# Supplementary material for: Healthcare worker fatigue during COVID-19, SARS, and MERS: a meta-analysis
Source: Occup Med (Lond). 2026 Mar 7;76(2):132–43. doi: 10.1093/occmed/kqag015 (PMC13095218; doi:10.1093/occmed/kqag015)
Supplement: kqag015_Supplementary_Data [file kqag015_supplementary_data.zip › 25-OP-062.R2 Supplementary Document S1 PRISMA Protocol FINAL.doc]

**Supplementary Document S1**

**TITLE:	PRISMA-P Protocol for a Systematic Review: Healthcare worker fatigue during COVID-19, SARS and MERS: A meta-analysis**

**REGISTRATION**:	PROSPERO No. CRD42021259045

**AUTHORS**:	Kim Poole-Wright	King’s College London

Harry Woodhall	King’s College London

Professor Trudie Chalder	King’s College London

**CONTACT**:	Trudie Chalder

IOPPN, King’s College London

De Crespigny Park

London

SE5 8AB

**EMAIL:	Trudie.chalder@kcl.ac.uk**

**CONTRIBUTIONS**:	Kim Poole-Wright	1^st^ Reviewer

Harry Woodhall	2^nd^ Reviewer

Professor Trudie Chalder	Senior Reviewer

**AMENDMENTS**:	Protocol amendments will be tracked, dated and numbered. The responsibility for tracking and registering changes to the protocol will be held by the 1^st^ Reviewer with prior agreement and approval from the Senior Reviewer. Final authorisation for any changes to the protocol will be from the Senior Reviewer.

A summary of changes log (Table 1, Appendix A.) will be used to monitor changes to the protocol and record authorisations. An explanation and rationale for the amendments will be logged in Table 2 (Appendix A.)

**FUNDING**:	No specific funding has been obtained for this review.

This protocol was developed and designed in collaboration between all stated authors.

**RATIONALE:**	The SARS-CoV-2 (COVID-19) pandemic has presented numerous challenges to healthcare systems and care providers. Critical care units worldwide treated people with potentially life-threatening symptoms and tackled the burden of care from large numbers of patients. These factors, combined with the psychological impact of care for critically ill patients, had a deleterious effect on those working on the frontline during COVID-19 (Restauri & Sheridan, 2020). Healthcare workers’ (HCW) risk for fatigue outcomes while deployed in pandemic affected hospitals have been found in a number of studies(Cai et al., 2020; Teng et al., 2020) and burnout is common (Bakken & Winn, 2021).

Fatigue outcomes during the earlier Severe Acute Respiratory Syndrome (SARS) pandemic were also found in some studies. For instance, in a study of SARS affected HCW in a Taiwan hospital, frontline nurses experienced significantly more exhaustion than administrative staff (Bai et al., 2004). Studies in Hong Kong and Korea found very high rates of fatigue, (71% and 79%) in staff following SARS (Chua et al., 2004) and MERS (Seo et al., 2020). Greater burnout symptoms were also reported in different settings and by different authors (Galanis et al., 2021; Kamali et al., 2022).

Contributors to fatigue have been found to be poor job control, prolonged stress, working patterns and workload. Working hours and night shifts, for instance, predicted fatigue in 2,667 nurses in China (Zhan et al., 2022). Furthermore, stress was associated with emotional or physical exhaustion among frontline medical staff during COVID-19 (Cai et al., 2020; Hu et al., 2020) and fear of being infected has been associated with exhaustion (Zerbini et al., 2020). Other factors implicated in fatigue include post-traumatic stress disorder (PTSD), anxiety and depression, which were associated with emotional exhaustion in Spanish HCW (Luceno-Moreno et al., 2022).

In summary, previous studies investigating COVID-19, SARS and Middle East Respiratory Syndrome (MERS) public health emergencies have indicated that HCW experience high levels of fatigue and exhaustion. Contributors to fatigue outcomes were depression, anxiety and PTSD, as well as stress. Working conditions such as shift patterns, and long hours have also shown to be associated. The aim of this review is to investigate fatigue outcomes in healthcare workers arising in the context of a coronavirus pandemic. In addition, this study will investigate potential predictors associated with fatigue.

**OBJECTIVES:**	The objectives of this review are: (a) to examine rates of fatigue among healthcare workers who worked during any of the named coronavirus pandemic or epidemic, (b) investigate potential predictors associated with fatigue outcomes (e.g. depression, anxiety, PTSD, job role, shifts etc), (c) synthesise the collected available data to conduct a meta-analysis (where appropriate). The study objectives will utilise a PICO framework (Appendix B.)

**METHODS**:	*Eligibility Criteria*

- Original articles available in English.
- Articles with primary data.
- Studies reporting fatigue or fatigue syndromes using a valid fatigue measure, a fatigue subscale or fatigue items on a questionnaire.
- Studies investigating HCW populations (defined as doctors, nurses, healthcare technicians/workers, porters, administration, paramedics, physical/psychological therapists, administrative staff etc) in the context of COVID-19, SARS and MERS.
- Any study design including cohort, case-control, cross-sectional, randomised controlled trials.

*Exclusion criteria*

- Compassion fatigue.
- Pandemic fatigue (defined as ‘worn out’ by pandemic warnings, or by government safety instructions, or with media coverage, or with compliance requirements’).
- Studies examining ‘emotional exhaustion’ forming part of ‘burnout syndrome’.
- Newspaper articles, conference papers, editorials, letters, opinions.
- Treatment procedures or protocols, qualitative studies, case reports, vaccination studies, ‘transmission’ studies, systematic or narrative reviews and meta-analyses.
- Absence of outcome data (i.e. not quantified or reported).
- HCW infected with a named virus.

*Information sources:*

PsycINFO, MEDLINE, EMBASE, CINAHL, OVID-HMIC, Cochrane Library

*Search Strategy:*

The search strategy will be piloted and amended where appropriate to select the most appropriate studies. An example of the search strategy is available in Appendix C. The search strategy language will be amended according to each database requirements.

*Study Records:*

The following data will be extracted and recorded in a spreadsheet: citation, population, sample size, follow-up period(s), location, inclusion & exclusion criteria, study design, study objectives, outcomes of interest, scales/instruments employed, comparator/intervention, results, effect size, power calculation conducted. In addition, the quality of each study (see Risk of Bias) will be indicated. A separate spreadsheet will be compiled detailing the studies that will be fully screened but excluded, together with the rationalisation for the exclusion.

*Selection Process:*

One reviewer will conduct the initial search in the selected databases for relevant studies. Two reviewers will identify studies based on the inclusion and exclusion criteria and extract data for each paper according to the protocol. The senior reviewer will independently screen a proportion of the selected studies and review the data extraction spreadsheet. Agreement for the final included studies for any meta-analysis or narrative review will be in collaboration with all reviewers. Disagreements will be settled through consensus and agreement. The second spreadsheet detailing the excluded fully screened papers will be reviewed by the senior researcher.

A PRISMA flow chart will be used to record the number of records collected, number of fully screened, records, number of records excluded, studies identified through reference lists or other sources and total number of records for inclusion in any meta-analysis.

*Data items/collection:*

The variables for the data to be recorded will include the following and will be entered into a data extraction spreadsheet:

- citation details
- target population (survivors, healthcare workers, general population, region/country),
- study eligibility criteria,
- population characteristics (socio-demographics)
- outcomes under study
- how the outcomes were measured (e.g. Chalder Fatigue Scale) including the definition of clinical outcomes for each scale, cut-off points, upper & lower scores, explanation of whether a high or low score is favourable
- study variables (e.g. PTSD, depression, working hours),
- metrics (e.g. changes in fatigue),
- timing of outcome measurements (e.g. assessments at 6-week intervals)
- mean and standard deviations for each group
- comparator group
- effect size,
- time (baseline data and follow-up times e.g. 1 month, 3 months),
- study design and setting (e.g. hospital, outpatients, population),
- context (e.g. SARS, MERS)
- study methods (single, multicentre, parallel, cluster)

*For randomised controlled trials:*

- Intervention or comparator descriptions (e.g. drug type, control group, placebo group)
- Doses, times and frequencies, length of intervention,
- How an intervention was assessed, length of exposure, cumulative exposure
- Integrity of the intervention (the degree to which the procedures were implemented as stated/planned)
- Post-intervention metrics (e.g. changes in fatigue, pre-post-test)
- Randomisation procedures
- Adverse effects

*Results*

- Number of participants in each stated group (including number of patients lost, withdrawn, lost to follow-up or excluded with reasons)
- Summary data for each group, each outcome and each time point (means and standard deviations for continuous data, OR for dichotomous data),
- Between-group estimates measuring effect of the intervention on the outcome (e.g. OR, RR, mean differences) and their confidence intervals
- Confounders measured

In the event of incomplete data relating to the outcomes, exposure, effect sizes, results or other important data, reviewers will request this information from the authors. Where there is no response, the missing data will be calculated according to Higgins (Higgins, 2003) or the paper will be excluded.

*Risk of bias:*

Risk of bias (RoB) assessment will be conducted for each included study using JBI Critical Appraisal Checklist for RCT and Quasi-experimental designs (JBI, 2022). The National Institutes of Health (NIH) Quality Assessment Tool was used for Observational Cohort and Cross-sectional designs (National Heart Lung and Blood Institute, 2021). RoB assessments will be reported in the data collection spreadsheet. The RoB will be conducted independently by one reviewer and checked for accuracy by the 2^nd^ reviewer. The assessments (e.g. good quality, fair quality, poor quality) will be reported. A selection of reviews will be independently assessed by the senior reviewer. Methods to summarise the RoB assessments for all the studies and a description of how these assessments will be incorporated into the data synthesis (i.e. sensitivity analyses) and their potential influence on the findings will be discussed.

*Data synthesis*

This systematic review will employ a quantitative approach and provide a summary pooled estimate of the risk for a fatigue outcome per exposure (virus type), combining the results of all the studies where appropriate. Where 3 or more studies can be combined based on the same outcome measure, a meta-analysis will be performed. Where there are less than 3 studies identified for the same outcome, the effect sizes will be described in text.

For the meta-analysis, odds ratios (OR) will be computed for binary outcomes (fatigued vs not fatigued) to estimate the risk of fatigue relative to each exposure virus and the target population, with 95% confidence intervals as an overall synthesised measure of effect size. For continuous outcomes, standardised mean differences for the combined effect size will be computed. Data from all relevant studies will be included in the analysis. Additional statistical tests may be conducted dependent upon data availability (e.g. fatigue outcome associated with gender, occupation type, comorbidities etc).

It is expected that there will be considerable heterogeneity in study types and outcome measures, therefore a random effects model will be performed for the meta-analysis to provide an estimate of the mean effect size for the included studies. The random effects model is expected to allow for wider heterogeneity and take account of the estimated between-study weight differences. To assess between-study-heterogeneity a Cochran’s Q will be performed, and the effect of heterogeneity will be quantified using the I^2^ statistical-test. A value of 50% or greater for the I^2^ will be considered as indicative of greater variability. A value of greater than 75% will be considered as considerable variability. Statistical measures of effect will be extracted from the included studies for calculating pooled effect sizes of the association between an included virus and fatigue outcomes. Effect sizes, 95% confidence intervals and statistical significance will be presented by quantitative and graphical representations (i.e. forest plots). Statistical significance will be set at p < 0.05 (2-tailed) for all analyses. Sensitivity analysis will be conducted utilising the quality assessments across all the studies. For example, excluding low grade studies, studies with declared conflicts of interest.

*Meta-bias(es)*

In order to analyse potential publication bias, funnel plots (observed for >10 studies included in the meta-analysis) with an Egger’s test (Egger et al., 1997) to test asymmetry at alpha level 0.1 will be conducted.

*Confidence in cumulative evidence*

GRADE (Grading of Recommendations, Assessment, Development and Evaluation working group methodology) will be used to assess the quality of evidence for all outcomes.

The quality of evidence will be assessed for risk of bias, consistency, directness, precision and publication bias. Quality will be judged as high (further research is very unlikely to change our confidence in the estimate of effect), moderate (further research is likely to have an important impact on our confidence in the estimate of effect and may change the estimate), low (further research is very likely to have an important impact on our confidence in the estimate of effect and is likely to change the estimate) or very low (very uncertain about the estimate of effect)

*Reporting standards*

This systematic review will comply with the PRISMA-P guidelines (Preferred Reporting Items for Systematic Reviews and Meta-Analyses Protocol (Moher et al., 2009).

Appendix A

*Table 1. SUMMARY OF CHANGES TABLE*

| *Document* | *Protocol Version Number* | *Date* | *Authorisation* |
| --- | --- | --- | --- |
| Amendment No. |  |  |  |
| Amendment No. |  |  |  |
| Amendment No. |  |  |  |
| Amendment No. |  |  |  |
| Current Protocol | 1.01 | 14.08.23 |  |
| Original | 1.01 | 04.08.20 |  |

*Table 2. AMENDMENT RATIONALE*

| *Section Number/Heading* | *Description of Amendment* | *Rationale Summary* |
| --- | --- | --- |
|  |  |  |
|  |  |  |
|  |  |  |
|  |  |  |

Appendix B

PICOS

| **Patient/Population** | **Exposure** | **Comparison** | **Outcome** |
| --- | --- | --- | --- |
| Adults | Coronavirus | Where applicable | Fatigue |
| Healthcare workers  Healthcare practitioners  Doctor, general practitioner, physician, clinician, surgeon, nurse, midwife, paramedic, healthcare employee, staff, professional, provider, emergency medical technician | SARS  MERS  COVID-19  Coronavirus  Epidemic  Pandemic | Healthy controls  Non-treatment  Placebo  Treatment as usual | Fatigue  Chronic fatigue  Chronic fatigue syndrome (CFS)  Exhaustion  Exhausted  Tiredness  Tired  Lethargy |

Appendix C

Example Search Strategy

Fatigue* or tired* or exhaustion or exhausted or lethargy And Nurse* or doctor* or midwife or midwives or surgeon* or paramedic* or EMT or emergency medical technician or GP or general practitioner* or healthcare worker* or healthcare professional*) or HCW* or medical staff or physician* or surgeon* or clinician* And Severe acute respiratory syndrome or SARS or SARS-CoV* or coronavirus or SARS-CoV-2 or COVID-19 or Covid* or Covid19 or middle eastern respiratory syndrome or MERS

**References**

Bai, Y. M., Lin, C., Lin, C., Chen, J., Chue, C., & Chou, P. (2004). Survey of Stress Reactions Among Health Care Workers Involved With the SARS Outbreak. *Psychiatric Services, 55*(9), 1055–1057. 10.1176/appi.ps.55.9.1055

Bakken, B. K., & Winn, A. N. (2021). Clinician burnout during the COVID-19 pandemic before vaccine administration. *Journal of the American Pharmacists Association : JAPhA, 61*(5), e71–e77. 10.1016/j.japh.2021.04.009

Cai, H., Tu, B., Ma, J., Chen, L., Fu, L., Jiang, Y., & Zhuang, Q. (2020). Psychological Impact and Coping Strategies of Frontline Medical Staff in Hunan Between January and March 2020 During the Outbreak of Coronavirus Disease 2019 (COVID‑19) in Hubei, China. *Medical Science Monitor, 26*, e924171. 10.12659/MSM.924171

Chua, S. E., Cheung, V., Cheung, C., McAlonan, G. M., Wong, J. W., Cheung, E. P., Chan, M. T., Wong, M. M., Tang, S. W., Choy, K. M., Wong, M. K., Chu, C. M., & Tsang, K. W. (2004). Psychological Effects of the SARS Outbreak in Hong Kong on High-Risk Health Care Workers. *The Canadian Journal of Psychiatry, 49*(6), 391–393. 10.1177/070674370404900609

Egger, M., Smith, G. D., Schneider, M., & Minder, C. (1997). Bias in meta-analysis detected by a simple, graphical test. *Bmj, 315*(7109), 629–634. 10.1136/bmj.315.7109.629

Galanis, P., Vraka, I., Fragkou, D., Bilali, A., & Kaitelidou, D. (2021). Nurses' burnout and associated risk factors during the COVID-19 pandemic: A systematic review and meta-analysis. *Journal of Advanced Nursing, 77*(8), 3286–3302. 10.1111/jan.14839

Higgins, J. P. T. (2003). Measuring inconsistency in meta-analyses. *Bmj, 327*(7414), 557–560. 10.1136/bmj.327.7414.557

Hu, D., Kong, Y., Li, W., Han, Q., Zhang, X., Zhu, L. X., Wan, S. W., Liu, Z., Shen, Q., Yang, J., He, H., & Zhu, J. (2020). Frontline nurses’ burnout, anxiety, depression, and fear statuses and their associated factors during the COVID-19 outbreak in Wuhan, China: A large-scale cross-sectional study. *eClinicalMedicine, 24*10.1016/j.eclinm.2020.100424

JBI. (2022, *JBI Critical Appraisal Tools.* <https://jbi-global/critical-appraisal-tools>

Kamali, M., Azizi, M., Moosazadeh, M., Mehravaran, H., Ghasemian, R., Reskati, M. H., & Elyasi, F. (2022). Occupational burnout in Iranian health care workers during the COVID-19 pandemic. *BMC Psychiatry, 22*10.1186/s12888-022-04014-x

Luceno-Moreno, L., Talavera-Velasco, B., & Martin-Garcia, J. (2022). Predictors of burnout in female nurses during the COVID-19 pandemic. *International Journal of Nursing Practice, 28*(5), 1–10. 10.1111/ijn.13084

Moher, D., Liberati, A., Tetzlaff, J., Altman, D. G., & Group, a. t. P. (2009). Preferred Reporting Items for Systematic Reviews and Meta-Analyses: The PRISMA Statement. *Annals of Internal Medicine, 151*(4), 264–269. 10.7326/0003-4819-151-4-200908180-00135

National Heart Lung and Blood Institute. (2021). Study Quality Assessment Tools. <https://www.nhlbi.nih.gov/health-topics/study-quality-assessment-tools>

Restauri, N., & Sheridan, A. D. (2020). Burnout and Posttraumatic Stress Disorder in the Coronavirus Disease 2019 (COVID-19) Pandemic: Intersection, Impact, and Interventions. *Journal of the American College of Radiology, 17*(7), 921–926. 10.1016/j.jacr.2020.05.021

Seo, Y. E., Kim, H. C., Yoo, S. Y., Lee, K. U., Lee, H. W., & Lee, S. H. (2020). Factors Associated with Burnout among Healthcare Workers during an Outbreak of MERS. *Psychiatry Investigation, 17*(7), 674–680. 10.30773/pi.2020.0056

Teng, Z., Wei, Z., Qiu, Y., Tan, Y., Chen, J., Tang, H., Wu, H., Wu, R., & Huang, J. (2020). Psychological status and fatigue of frontline staff two months after the COVID-19 pandemic outbreak in China: A cross-sectional study. *Journal of Affective Disorders, 275*, 247–252. 10.1016/j.jad.2020.06.032

Zerbini, G., Ebigbo, A., Reicherts, P., Kunz, M., & Messman, H. (2020). Psychosocial burden of healthcare professionals in times of covid-19—a survey conducted at the university hospital Augsburg. *German Medical Science, 18*, Doc05. 10.3205/000281

Zhan, Y., Liu, Y., Chen, Y., Liu, H., Zhang, W., Yan, R., Yu, J., & Li, S. (2022). The prevalence and influencing factors for compassion fatigue among nurses in Fangcang shelter hospitals: A cross-sectional study. *International Journal of Nursing Practice, 28*(5), 1–12. 10.1111/ijn.13054
