# Supplementary material for: Healthcare worker fatigue during COVID-19, SARS, and MERS: a meta-analysis
Source: Occup Med (Lond). 2026 Mar 7;76(2):132–43. doi: 10.1093/occmed/kqag015 (PMC13095218; doi:10.1093/occmed/kqag015)
Supplement: kqag015_Supplementary_Data [file kqag015_supplementary_data.zip › 25-OP-062.R2 Supplementary document S7 References 71-123 FINAL.doc]

**Supplementary document S7 References 71-123**

71. Frenkel MO, Pollak KM, Schilling O*, et al.* Stressors faced by healthcare professionals and coping strategies during the early stage of the COVID-19 pandemic in Germany *PLoS One* 2022;**17**:e0261502.

72. Wu J, Li H, Geng Z, Wang Y, Wang X, Zhang J. Subtypes of nurses' mental workload and interaction patterns with fatigue and work engagement during coronavirus disease 2019 (COVID-19) outbreak: A latent class analysis *BMC nursing* 2021;**20**:206.

73. Cotrin P, Moura W, Gambardela-Tkacz CM*, et al.* Healthcare Workers in Brazil during the COVID-19 Pandemic: A Cross-Sectional Online Survey *Inquiry (Chicago)* 2020;**57**:46958020963711.

74. Sagherian K, Steege LM, Cobb SJ, Cho H. Insomnia, fatigue and psychosocial well-being during COVID-19 pandemic: A cross-sectional survey of hospital nursing staff in the United States *J Clin Nurs* 2023;**32**:5382-5395.

75. Jarrar Mt, Binti Ali N, Shahruddin R*, et al.* The Impact of the Working Hours Among Malaysian Nurses on Their Ill-Being, Intention to Leave, and the Perceived Quality of Care: A Cross-Sectional Study During the COVID-19 Pandemic *Journal of multidisciplinary healthcare* 2023;**16**:119-131.

76. Hwang S, Kwon KT, Lee SH*, et al.* Correlates of burnout among healthcare workers during the COVID-19 pandemic in South Korea *Scientific reports* 2023;**13**:3360.

77. Pi R, Liu Y, De Z*, et al.* The prevalence and factors of work-related fatigue among nurses: a meta-analysis and systematic review *Research Square* 2024.

78. Gates M, Wingert A, Featherstone R, Samuels C, Simon C, Dyson MP. Impact of fatigue and insufficient sleep on physician and patient outcomes: a systematic review *BMJ Open* 2018;**8**:e-e021967.

79. Gifkins J, Johnston A, Loudoun R, Troth A. Fatigue and recovery in shiftworking nurses: A scoping literature review *International journal of nursing studies* 2020;**112**:103710.

80. Vizheh M, Qorbani M, Arzaghi SM, Muhidin S, Javanmard Z, Esmaeili M. The mental health of healthcare workers in the COVID-19 pandemic: A systematic review *Journal of Diabetes & Metabolic Disorders* 2020;**19**:1967-1978.

81. Koontalay A, Suksatan W, Prabsangob K, Sadang JM. Healthcare Workers’ Burdens During the COVID-19 Pandemic: A Qualitative Systematic Review *Journal of multidisciplinary healthcare* 2021;**14**:3015-3025.

82. Rauch S, van Veelen MJ, Oberhammer R*, et al.* Effect of wearing personal protective equipment (PPE) on CPR quality in times of the COVID-19 pandemic—a simulation, randomised crossover trial *Journal of clinical medicine* 2021;**10**:1728.

83. Tabah A, Ramanan M, Laupland KB*, et al.* Personal protective equipment and intensive care unit healthcare worker safety in the COVID-19 era (PPE-SAFE): An international survey *Journal of Critical Care* 2020;**59**:70-75.

84. Agarwal A, Agarwal S, Motiani P. Difficulties Encountered While Using PPE Kits and How to Overcome Them: An Indian Perspective *Cureus* 2020;**12**:e11652.

85. Li F, Jiang T, Shi T*, et al.* Factors that affect the duration of wearing disposable personal protective equipment by healthcare professionals in Wuhan during treatment of COVID-19 patients: An epidemiological study *Nursing & health sciences* 2021;**23**:245-254.

86. Thiagarajan S, Shetty P, Gulia A, Prakash G, Pramesh CS, Puri A. A Survey of Personnel Protective equipment’s (PPE) Use and Comfort Levels Among Surgeons During Routine Cancer Surgery in the COVID-19 Pandemic *Indian journal of surgical oncology* 2021;**12**:365-373.

87. Karachalios T, Maasalu K, Fellander-Tsai L. Personal protection equipment for orthopaedic and trauma surgery during the COVID-19 pandemic: The results of an EFORT survey initiative *EFORT open reviews* 2022;**7**:122-128.

88. Zhang N, Xu D, Li J, Gong Z. Effect of Perceived Organizational Support on Fatigue of Chinese Nurses during the COVID-19 Pandemic: Resilience as a Mediator *The international journal of mental health promotion* 2021;**23**:243-254.

89. Yánez Benitez C, Güemes A, Aranda J*, et al.* Impact of Personal Protective Equipment on Surgical Performance During the COVID-19 Pandemic *World journal of surgery* 2020;**44**:2842-2847.

90. Cai H, Garcia A, Polivka B, Spreckelmeyer K, Yang FM. Visibility and Accessibility of Hand Hygiene Stations and Fatigue Among Nurses Working in Long-Term Care (LTC) During the COVID-19 Pandemic *HERD* 2023;**16**:24-37.

91. Zhou Y, Ding H, Zhang Y*, et al.* Prevalence of poor psychiatric status and sleep quality among frontline healthcare workers during and after the COVID-19 outbreak: a longitudinal study *Translational psychiatry* 2021;**11**:223.

92. Huffman EM, Athanasiadis DI, Anton NE*, et al.* How resilient is your team? Exploring healthcare providers’ well-being during the COVID-19 pandemic *The American journal of surgery* 2021;**221**:277-284.

93. McDonald G, Jackson D, Wilkes L, Vickers M. Personal resilience in nurses and midwives: Effects of a work-based educational intervention *Contemporary nurse : a journal for the Australian nursing profession* 2013;**45**:134-143.

94. Xu D, Zhang N, Bu X, Xu Z. A latent profile analysis of psychological resilience associated with work fatigue among Chinese nurses *Research in nursing & health* 2024;**47**:335-343.

95. Barker LM, Nussbaum MA. Fatigue, performance and the work environment: a survey of registered nurses *Journal of Advanced Nursing* 2011;**67**:1370-1382.

96. Christmas A, Mackinnon C, Brincat E, Donnelly P, Worrall M. Exploring the psychological impact of the COVID-19 pandemic on PICU consultants, advanced nurse practitioners and trainee doctors *British journal of child health* 2022;**3**:77-88.

97. Nazario EG, Silva RM, Beck CL, Centenaro AP, Freitas EO, Miranda FM. Fatigue and sleep in intensive care nursing workers in the COVID-19 pandemic 2023.

98. Akıncı T, Başar HM. Assessment of Sleep Quality in Healthcare Workers as Part of the COVID-19 Outbreak *Türk nöroloji dergisi* 2021;**27**:176-186.

99. Duong-Quy S, Tran-Duc S, Hoang-Chau-Bao D, Bui-Diem K, Vu-Tran-Thien Q, Nguyen-Nhu V. Tiredness, depression, and sleep disorders in frontline healthcare workers during COVID-19 pandemic in Vietnam: A field hospital study *Front Psychiatry* 2022;**13**:984658.

100. Zhan Y, Liu Y, Liu H*, et al.* Factors associated with insomnia among Chinese front-line nurses fighting against COVID-19 in Wuhan: A cross-sectional survey *Journal of nursing management* 2020;**28**:1525-1535.

101. Zhang Z, Hu Y, Chen Y, Liao Z, Zheng Y, Ding L. Sleep disorders and related factors among frontline medical staff supporting Wuhan during the COVID-19 outbreak *Bulletin of the Menninger Clinic* 2021;**85**:254-270.

102. Dai C, Hu P, Yan F*, et al.* The mediating role of sleep quality in the relationship between dispositional mindfulness and fatigue in Chinese nurses during the COVID-19 pandemic *BMC Nurs* 2023;**22**:471.

103. Demerouti E, Bakker AB, Nachreiner F, Schaufeli WB. The job demands-resources model of burnout *J Appl Psychol* 2001;**86**:499-512.

104. Finn R, Ganau M, Jenkinson MD, Plaha P. COVID-legal study: neurosurgeon experience in Britain during the first phase of the COVID-19 pandemic - medico-legal considerations *Br J Neurosurg* 2021;**35**:547-550.

105. Sagherian K, Clinton ME, Abu-Saad Huijer H, Geiger-Brown J. Fatigue, Work Schedules, and Perceived Performance in Bedside Care Nurses *Workplace Health Saf* 2017;**65**:304-312.

106. Adams JG, Walls RM. Supporting the Health Care Workforce During the COVID-19 Global Epidemic *JAMA* 2020;**323**:1439-1440.

107. Schaufeli WB. Applying the Job Demands-Resources model: A ‘how to’ guide to measuring and tackling work engagement and burnout *Organizational Dynamics* 2017;**46**:120-132.

108. Kunzler AM, Chmitorz A, Rothke N*, et al.* Interventions to foster resilience in nursing staff: A systematic review and meta-analyses of pre-pandemic evidence *Int J Nurs Stud* 2022;**134**:104312.

109. Smith B, Shatte A, Perlman A, Siers M, Lynch WD. Improvements in Resilience, Stress, and Somatic Symptoms Following Online Resilience Training: A Dose-Response Effect *J Occup Environ Med* 2018;**60**:1-5.

110. Steven A, Redfern N. Fatigue: For safe patients we need safe nurses *J Adv Nurs* 2025;**81**:5627-5631.

111. Sutherland C, Smallwood A, Wootten T, Redfern N. Fatigue and its impact on performance and health *Br J Hosp Med (Lond)* 2023;**84**:1-8.

112. Su C-Y, Peng C-Y, Liu H-L, Yeh IJ, Lee C-W. Comparison of Effects of N95 Respirators and Surgical Masks to Physiological and Psychological Health among Healthcare Workers: A Randomized Controlled Trial *International journal of environmental research and public health* 2021;**18**:13308.

113. Giordano F, Scarlata E, Baroni M*, et al.* Receptive music therapy to reduce stress and improve wellbeing in Italian clinical staff involved in COVID-19 pandemic: A preliminary study *The Arts in psychotherapy* 2020;**70**:101688.

114. Bektas Akpinar N, Ozcan Yuce U, Yurtsever S. The Effect of Distant Reiki on the Stress and Fatigue Levels of Nurses Working in COVID-19 Clinics: A Randomized-Controlled, Single-Blind Study *Holist Nurs Pract* 2024;**38**:102-108.

115. Karimi N, Hasanvand S, Beiranvand A, Gholami M, Birjandi M. The effect of Aromatherapy with Pelargonium graveolens (P. graveolens) on the fatigue and sleep quality of critical care nurses during the Covid-19 pandemic: A randomized controlled trial *Explore (New York, NY)* 2024;**20**:82-88.

116. Baek Y, Han K, Kim J, Yoo HY. Smartphone-based home workout program for shift-work nurses working during the COVID-19 pandemic *Nurs Health Sci* 2022;**24**:708-716.

117. Ha Y, Lee S-H, Lee D-H, Kang Y-H, Choi W, An J. Effectiveness of a Mobile Wellness Program for Nurses with Rotating Shifts during COVID-19 Pandemic: A Pilot Cluster-Randomized Trial *International journal of environmental research and public health* 2022;**19**.

118. Ma M, Adeney M, Long H, He B. The Environmental Factors Associated With Fatigue of Frontline Nurses in the Infection Disease Nursing Unit *Frontiers in public health* 2021;**9**:774553.

119. Ulrich RS, Zimring C, Zhu X*, et al.* A Review of the Research Literature on Evidence-Based Healthcare Design *HERD* 2008;**1**:61-125.

120. Stuijfzand S, Deforges C, Sandoz V*, et al.* Psychological impact of an epidemic/pandemic on the mental health of healthcare professionals: a rapid review *BMC Public Health* 2020;**20**:1230.

121. Muller AE, Hafstad EV, Himmels JPW*, et al.* The mental health impact of the covid-19 pandemic on healthcare workers, and interventions to help them: A rapid systematic review *Psychiatry Research* 2020;**293**:113441.

122. Alnazly E, Khraisat OM, Al-Bashaireh AM, Bryant CL. Anxiety, depression, stress, fear and social support during COVID-19 pandemic among Jordanian healthcare workers *PLoS One* 2021;**16**:e0247679.

123. Shen X, Zou X, Zhong X, Yan J, Li L. Psychological stress of ICU nurses in the time of COVID-19 *Critical Care* 2020;**24**:200.
