# Supplementary material for: Healthcare worker fatigue during COVID-19, SARS, and MERS: a meta-analysis
Source: Occup Med (Lond). 2026 Mar 7;76(2):132–43. doi: 10.1093/occmed/kqag015 (PMC13095218; doi:10.1093/occmed/kqag015)
Supplement: kqag015_Supplementary_Data [file kqag015_supplementary_data.zip › 25-OP-062.R2 Supplementary Figure S5 Funnel plot for fatigue prevalence analysis FINAL.doc]

Supplementary S5 Funnel plots of fatigue prevalence

**Funnel plot for fatigue in healthcare workers (n.32)**

**Funnel plot for fatigue related to wearing PPE**
