# Supplementary material for: Healthcare worker fatigue during COVID-19, SARS, and MERS: a meta-analysis
Source: Occup Med (Lond). 2026 Mar 7;76(2):132–43. doi: 10.1093/occmed/kqag015 (PMC13095218; doi:10.1093/occmed/kqag015)
Supplement: kqag015_Supplementary_Data [file kqag015_supplementary_data.zip › 25-OP-062.R2 Supplementary S2 Full Search Results FINAL.doc]

Supplementary document S2

Full Search Results

Embase Classic+Embase <1947 to 2024 July 05>

1	(fatigue* or tired* or exhaustion or exhausted or lethargy).mp. [mp=title, abstract, heading word, drug trade name, original title, device manufacturer, drug manufacturer, device trade name, keyword heading word, floating subheading word, candidate term word]	473357

2	(nurse* or doctor* or midwife or midwives or surgeon* or paramedic* or EMT or GP or general practitioner*).mp. [mp=title, abstract, heading word, drug trade name, original title, device manufacturer, drug manufacturer, device trade name, keyword heading word, floating subheading word, candidate term word]	1593862

3	(((((healthcare adj2 worker*) or healthcare) adj2 professional*) or HCW* or medical) adj2 staff).mp. [mp=title, abstract, heading word, drug trade name, original title, device manufacturer, drug manufacturer, device trade name, keyword heading word, floating subheading word, candidate term word]	60073

4	(physician* or surgeon* or clinician*).mp. [mp=title, abstract, heading word, drug trade name, original title, device manufacturer, drug manufacturer, device trade name, keyword heading word, floating subheading word, candidate term word]	1770221

5	("Severe acute respiratory syndrome" or SARS or SARS-CoV).mp. [mp=title, abstract, heading word, drug trade name, original title, device manufacturer, drug manufacturer, device trade name, keyword heading word, floating subheading word, candidate term word]	232049

6	("middle eastern respiratory syndrome" or MERS).mp. [mp=title, abstract, heading word, drug trade name, original title, device manufacturer, drug manufacturer, device trade name, keyword heading word, floating subheading word, candidate term word]	9667

7	(coronavirus or SARS-CoV-2 or COVID-19 or Covid* or Covid19).mp. [mp=title, abstract, heading word, drug trade name, original title, device manufacturer, drug manufacturer, device trade name, keyword heading word, floating subheading word, candidate term word]	555871

8	2 or 3 or 4	2671010

9	5 or 6 or 7	568632

10	1 and 8 and 9	3384

APA PsycInfo <1806 to July Week 1 2024>

1	(fatigue* or tired* or exhaustion or exhausted or lethargy).mp. [mp=title, abstract, heading word, table of contents, key concepts, original title, tests & measures, mesh word]	48983

2	(nurse* or doctor* or midwife or midwives or surgeon* or paramedic* or EMT or GP or general practitioner*).mp. [mp=title, abstract, heading word, table of contents, key concepts, original title, tests & measures, mesh word]	151570

3	(((((healthcare adj2 worker*) or healthcare) adj2 professional*) or HCW* or medical) adj2 staff).mp. [mp=title, abstract, heading word, table of contents, key concepts, original title, tests & measures, mesh word]	4373

4	(physician* or surgeon* or clinician*).mp. [mp=title, abstract, heading word, table of contents, key concepts, original title, tests & measures, mesh word]	209566

5	("Severe acute respiratory syndrome" or SARS or SARS-CoV).mp. [mp=title, abstract, heading word, table of contents, key concepts, original title, tests & measures, mesh word]	7856

6	("middle eastern respiratory syndrome" or MERS).mp. [mp=title, abstract, heading word, table of contents, key concepts, original title, tests & measures, mesh word]	213

7	(coronavirus or SARS-CoV-2 or COVID-19 or Covid* or Covid19).mp. [mp=title, abstract, heading word, table of contents, key concepts, original title, tests & measures, mesh word]	51186

8	2 or 3 or 4	329211

9	5 or 6 or 7	51847

10	1 and 8 and 9	358

OVID-HMIC Health Management Information Consortium <1979 to JULY 2024>

1	(fatigue* or tired* or exhaustion or exhausted or lethargy or asthenia).mp. [mp=title, other title, abstract, heading words]	1004

2	(nurse* or doctor* or midwife or midwives or surgeon* or paramedic* or EMT or GP or general practitioner* or physician).mp. [mp=title, other title, abstract, heading words]	67915

3	(((((healthcare adj2 worker*) or healthcare) adj2 professional*) or HCW* or medical) adj2 staff) or healthcare) adj2 employee) or healthcare) adj2 provider).mp. [mp=title, other title, abstract, heading words]	172

4	(physician* or surgeon* or clinician*).mp. [mp=title, other title, abstract, heading words]	14736

5	("Severe acute respiratory syndrome" or SARS or SARS-CoV).mp. [mp=title, other title, abstract, heading words]	321

6	("middle eastern respiratory syndrome" or MERS).mp. [mp=title, other title, abstract, heading words]	8

7	(coronavirus or SARS-CoV-2 or COVID-19 or Covid* or Covid19).mp. [mp=title, other title, abstract, heading words]	3210

8	2 or 3 or 4	75181

9	5 or 6 or 7	3429

10	8 and 9	4

Ovid MEDLINE(R) ALL <1946 to July 05, 2024>

1	(fatigue* or tired* or exhaustion or exhausted or lethargy).mp. [mp=title, book title, abstract, original title, name of substance word, subject heading word, floating sub-heading word, keyword heading word, organism supplementary concept word, protocol supplementary concept word, rare disease supplementary concept word, unique identifier, synonyms, population supplementary concept word, anatomy supplementary concept word]	197171

2	(nurse* or doctor* or midwife or midwives or surgeon* or paramedic* or EMT or GP or general practitioner*).mp. [mp=title, book title, abstract, original title, name of substance word, subject heading word, floating sub-heading word, keyword heading word, organism supplementary concept word, protocol supplementary concept word, rare disease supplementary concept word, unique identifier, synonyms, population supplementary concept word, anatomy supplementary concept word]	968298

3	(((((healthcare adj2 worker*) or healthcare) adj2 professional*) or HCW* or medical) adj2 staff).mp. [mp=title, book title, abstract, original title, name of substance word, subject heading word, floating sub-heading word, keyword heading word, organism supplementary concept word, protocol supplementary concept word, rare disease supplementary concept word, unique identifier, synonyms, population supplementary concept word, anatomy supplementary concept word]	43465

4	(physician* or surgeon* or clinician*).mp. [mp=title, book title, abstract, original title, name of substance word, subject heading word, floating sub-heading word, keyword heading word, organism supplementary concept word, protocol supplementary concept word, rare disease supplementary concept word, unique identifier, synonyms, population supplementary concept word, anatomy supplementary concept word]	1212495

5	("Severe acute respiratory syndrome" or SARS or SARS-CoV).mp. [mp=title, book title, abstract, original title, name of substance word, subject heading word, floating sub-heading word, keyword heading word, organism supplementary concept word, protocol supplementary concept word, rare disease supplementary concept word, unique identifier, synonyms, population supplementary concept word, anatomy supplementary concept word]	247068

6	("middle eastern respiratory syndrome" or MERS).mp. [mp=title, book title, abstract, original title, name of substance word, subject heading word, floating sub-heading word, keyword heading word, organism supplementary concept word, protocol supplementary concept word, rare disease supplementary concept word, unique identifier, synonyms, population supplementary concept word, anatomy supplementary concept word]	8737

7	(coronavirus or SARS-CoV-2 or COVID-19 or Covid* or Covid19).mp. [mp=title, book title, abstract, original title, name of substance word, subject heading word, floating sub-heading word, keyword heading word, organism supplementary concept word, protocol supplementary concept word, rare disease supplementary concept word, unique identifier, synonyms, population supplementary concept word, anatomy supplementary concept word]	450552

8	2 or 3 or 4	1804376

9	5 or 6 or 7	460494

10	1 and 8 and 9	1545

CINAHL

S1	MW (SARS OR severe acute respiratory syndrome OR SARS* OR SARS-CoV-2 OR COVID-19 OR Covid OR coronavirus OR COVID19 OR SARS-CoV OR SARS-CoV* OR SARSCOV2 OR SARSCOV-2 OR nCoV-2 OR nCoV19* OR nCoV2 or MERS or Middle Eastern respiratory syndrome) 106,186

S2	MH (fatigue OR exhaustion OR tiredness OR tired OR lethargy OR asthenia OR exhaust*) 23,500

S3	MH (chronic fatigue OR chronic fatigue syndrome) 12,451

S4	S2 OR S3 35,825

S5	S1 AND S4 NOT vaccine NOT vaccination NOT Covid syndrome NOT children NOT adolescents NOT symptom* Expanders – apply equivalent subjects 293

Cochrane Library

#1	(lethargy):ti,ab,kw OR (asthenia or fatigue): ti,ab,kw OR (chronic fatigue):ti,ab,kw 51917

#2	nurse OR doctor OR EMT OR paramedic OR physician OR surgeon OR healthcare employee OR healthcare worker OR healthcare professional OR healthcare practitioner OR healthcare staff 107632

#3	exhaust* OR tired OR tiredness9276

#4	general practitioner OR clinician 25647

#5	MeSH descriptor: [coronavirus infections] this item only 867

#6	MeSH descriptor: [SARS-CoV-2] explode all trees 3484

#7	MERS OR SARS OR covid OR COVID-19 OR COVID19 OR SARS-CoV OR SARS-COV NOT long-covid 22712

#8	#1 OR #3 59129

#9	#2 OR #4 125889

#10	#5 OR #6 OR #7 22746

#11	#8 AND #9 AND #10 488 (296 trials)
