# Supplementary material for: Healthcare worker fatigue during COVID-19, SARS, and MERS: a meta-analysis
Source: Occup Med (Lond). 2026 Mar 7;76(2):132–43. doi: 10.1093/occmed/kqag015 (PMC13095218; doi:10.1093/occmed/kqag015)
Supplement: kqag015_Supplementary_Data [file kqag015_supplementary_data.zip › 25-OP-062.R2 Supplementary Table S3 Summary Table for All Included Studies FINAL.doc]

Supplementary S3 Summary table of all included studies (n=88)

| **Author (year)**  **Country** | **Study Design** | **Data**  **Collected** | **N** | **Profession** | **Scale** | **Fatigue Outcome** | **Fatigue prevalence**  **N (%)** | **Associations with Fatigue** | **Associations Results**  **N (%)** | **Controls**  **N (%)** | **p** |
| --- | --- | --- | --- | --- | --- | --- | --- | --- | --- | --- | --- |
| Argawal et al. (2020)  India | Cohort  Survey | July 2020 | 253 | Doctors  Nurses  Technical  Ancillary | ADQ | Fatigue | **-** | PPE related fatigue | 190 (75) | - | - |
| Akinci et al. (2021) | Cross-sectional | April -  May 2020. | 152 | HCW | FSS | Fatigue | **-** | **Sleep quality**  *PSQI Score =<5*  FSS ≤6.2  FSS ≥6.2  *PSQI Score =>5*  FSS ≤6.2  FSS ≥6.2 | 3 (7)  40 (93)  10 (9)  96 (91) |  | .652 |
| Ampil et al. (2022) | Cross-sectional  Survey | January 2021 - August 2021 | 1349 | Doctors  Nurses  Technical  Others | ADQ | Exhaustion | Physical  175 (13.0)  Mental  188 (13.9) | Higher hesitation to work  Physical exhaustion  Lower hesitation to work  Mental exhaustion | aOR 19.56  aOR 0.35 |  | .002  .03 |
| Asaoka et al. (2020)  Japan | Cross-sectional  Survey | March – April 2020 | 331 | DMAT*  DPAT* | SRQ | Exhaustion | - | Post-traumatic stress symptoms | *β =*1.59 | - | .001 |
| Baek et al. (2022)  South Korea | Quasi-experiment | - | 49 | Nurses | MFS | Fatigue | - | **Smartphone home workout intervention**  Baseline  12 weeks  18 weeks | **M (SD)**  92.0 (15.9)  83.8 (17.4)  81.8 (16.5) | **M (SD)**  95.0 (14.8)  94.8 (15.0)  97.7 (16.2) | .02 |
| Barpanda & Saraswathy (2023)  India | Cross-sectional | NR | 238 | HCW | FSS | Fatigue | M(SD)  29.90 (15.74) | Quality of work life  Sleeping disorders  Department with fatigue  Age with fatigue  Marital status with fatigue  Gender | r=−0.068  r =0.261  F=10.660  F=4.043  F=7.150  T=2.258 |  | >.05  0.01  .000  .019  .008  >.05 |
| Bektaş Akpınar et al. (2024)  Turkey | RCT | May, July 2021 | 62 | Nurses | VAS-F | Fatigue | - | **Reiki intervention**  Pretest  Post test | **M (SD)**  5.37 (0.22)  4.11 (0.11) | **M (SD)**  5.12 (0.25)  5.52 (0.26) | .01  .119 |
| Bodnar et al. (2024)  Australia | Quasi-experiment  PPE | February -June 2021 | 63 | Doctors  Nurses | SamnPerelli | Fatigue | - | Postural symptoms before/after shift  Night v Day shift  Afternoon vs Day shift  HRZ v Non-HRZ  Hours between sampling  Gender  Age | aOR 3.12  aOR 1.60  aOR 5.16  aOR 0.23  aOR 0.97  aOR 1.22  aOR 0.97 |  |  |
| Budrevičiūtė et al. (2022)  Lithuania | Cross-sectional | June -September 2021 | 191 | Physicians | ADQ | Fatigue | 145 (75.9) | *Urban/Rural location*  Consultations  Patients’ fears of attending in-person consultations  *Men vs Women*  Chaotic vaccination priorities  *Age* | χ^2^=9.974  χ^2^=9.45  χ^2^= 6.69  **-** |  | .041  .009  .035  .002 |
| Cai et al. (2023)  USA | Cross-sectional | June 2020, January 2021 | 297 | Nurses | PROMIS | Fatigue | 233 (78.3) | Hand hygiene design  *Age*  *Licensed/registered nurses*  *Direct patient care*  *Non-profit/profit*  *Urban/rural location*  *Education level*  Care for patients (guilt)  Putting family at risk (guilt)  Organisational support  Confident about future | OR 0.37  OR 0.78  OR 1.08  OR 0.82  OR 0.85  OR 1.04  OR 0.85  OR 1.02  OR 1.46  OR 0.97  OR 0.94 | - | .002  .388  .833  .433  .539  .895  .186  .876  .012  .826  .739 |
| Caliskan et al. (2022)  Turkey | Cross-sectional  Survey | October – December 2020 | 538 | Nurses  Doctors  Midwives  Others | OFER | Fatigue  Chronic | **M (SD)**  71,94 (26,67) | **Chronic fatigue**  Work unit (ICU)  Increased work time  Children at home  Profession  Working hours  Gender  Has chronic disease  Marital status  No. of children  With older relative at home  Age  Virus phobia  Acute fatigue  Recovery Dimension (OFER) | F =3,626  F =26,214  F =2,863  F =5,463  F =1,815  t =-1,435  t =3,626  t = 0,257  F 0,798  t = 0,359  r =-.044  r =.590  r =.760  r =-.713 |  | .003  .000  .004  .001  .164  .152  .003  .797  .495  .720  NS  <.01  <.01  <.01 |
| Chen et al. (2023)  China | Cross-sectional  Survey | August 2022 | 514 | Nurses | FSS | Fatigue | 296 (57.59) | Mindfulness scores  Gender  Education, Age, Children, Title  Marital status (Married)  Years working (>15)  Job seniority  Sleep (≤5, 5-8, >8 hrs)  Somatic symptoms  Adapting to dietary habit | β =0.94  β =0.33  -  β =0.21  β =0.09  β =1.68  β =1.15  -  β =1.70 |  | <.001  .036  NS  .003  .013  .477  .461  NS  .012 |
| Cho et al. (2023)  South Korea | Cross-sectional  Survey | May, July 2021 | 198 | Nurses | MAF | Fatigue | **M (SD)**  28.51 (9.72) | Age  Employment type  Occupational stress  Anxiety  Depression  Sleep disturbance | β =−0.14  β =2.53  β =0.23  β =0.43  β =0.33  β =0.72 |  | .004  .017  <.001  .016  .043  <.001 |
| Christmas et al. (2022)  UK | Cross-sectional Survey | May – June 2020 | 18 | Doctors  Nurses | ADQ | Fatigue | 14/17 (82.4) | - | - | - | - |
| Chua et al. (2004)  Hong Kong | Cross-sectional  SARS | April – May 2003 | 613 | Nurses  Doctors  Others | ADQ | Tiredness | 192 (71) | - | - | - | - |
| Costa et al. (2021)  Italy | Cross-sectional  Survey | February -August 2020 | 119 | Doctors | POMS | Fatigue | **M (SD)**  10.95 (4.66) | Fatigue between professions  Year of residency  *Anaesthesiology*  *Service*  *Surgical*  *Night shifts (Service)*  *Night shifts (Surgical)* | -  β =−2.51  β =−1.63  β =−2.96  β =−2.84  β =6.42 | - | 0.512  .003  .003  .002  .037  .009 |
| Cotrin et al., (2020)  Brazil | Cross-sectional  Survey | - | 536 | Physicians  Nurses  Dentists | ADQ | Tired | 373 (69.5) | - | - | - | - |
| Dai et al. (2023)  China | Cross-sectional  Survey | August – September 2022 | 2143 | Nurses | FS-14 | Fatigue | **M (SD)**  8.17 (3.70) | Mindfulness  Sleep quality  Fatigue & mindfulness mediated by sleep quality | r =-0.518  r =0.547  β =-0.374 |  | <.001  <.001  <.001 |
| de Cordova et al. (2022)  USA | Cross-sectional  Survey | October 2020 | 3030 | Nurses | NRS | Exhaustion | 2426 (80.1) | PPE confidence  Intention to leave | OR =3.89  X^2^ =57.8 |  | .05 |
| Duong-Quy et al. (2022)  Vietnam | Cross-sectional | - | 100 | HCW | Pichot’s Fatigue Scale | Fatigue | **M (SD)**  4.18 (5.42) | Sleep apnoea  Sleep latency  Snoring | t= -2.33  t=2.08  t=-0.77 |  | ,002  .02  .02 |
| Engelmann et al. (2023)  Germany | Prospective cohort | April, August 2020 | 1185 | Doctors  Nurses  Others | NRS | Fatigue | **M (SD)**  T0=3.82 (2.53)  T1=4.16 (2.61) | Between T0-T1  Somatic symptom burden | t=-3.85  β =0.12 |  | .001  .002 |
| Erdagi Oral et al. (2022)  Turkey | Cross-sectional | September, December 2020 | 460 | Nurses | CFQ | Fatigue  Total  Physical  Mental | **M (SD)**  20.14 (6.72)  13.93 (4.55)  6.21 (2.79) | **PPE Effects**  *Allergic reaction*  *Facial redness/sores*  *Wounds behind ears*  *Scalp itching*  *Lower limb oedema*  *Headaches/dizziness*  *Forehead wounds*  *Falling*  *Bladder pain*  *Increased needle/sharp injuries*  *Hoarseness*  *Hearing problems*  *Sweating* | t =5.051  t =8.988  t =6.222  t =8.629  t =6.379  t =5.949  t =5.076  t =5.562  t =5.732  t =6.025  t =8.173  t =5.758  t =3.422 |  | .000  .000  .000  .000  .000  .000  .000  .000  .000  .000  .000  .000  .001 |
| Finn et al. (2021)  UK | Cross-sectional  Survey | June 2020 | 106 | Surgeons | ADQ | Fatigue | - | PPE | 66 (62) | - | - |
| Franco et al. (2020)  Ecuador | Cross-sectional  Survey | March – May 2020 | 127 | Nurses | ADQ | Fatigue | 85 (66.9) | - | - | - | - |
| Frenkel et al. (2022)  Germany | Cross-sectional  Survey | April – June 2020 | 575 | HCW | ADQ | Fatigue | - | Workload interfering with private life  Concern for team  Fear of transmission  Uncertainty/lack of knowledge  Between sector/location fatigue | β =-0.51  β =-0.190  β =0.048  β = 0.059  W(2) = 14.37 | - | <.001  <.001  .291  .054  <.001 |
| Giordano et al. (2020)  Italy | Quasi-experiment  Pilot study | April – May 2020 | 34 | Nurses  Doctors | MTC-Q1 | Tiredness | - | Breathing playlist (T0-T1)  Energy playlist (T0-T1)  Customised playlist (T0-T1)  Customised & Serenity playlists | t =7,695  t =4,873  t =10,423  - |  | <.05  <.05  .000  NS |
| Grelier et al. (2022)  France | Cross-sectional  Survey | May – June 2020 | 794 | Nurses  Physicians | ADQ | Fatigue | **M (SD)**  6.2 (-) | Working in a Covid unit | - | - | .014 |
| Gupta et al. (2021)  India | Cross-sectional | April – July 2020 | 353 | HCW | ADQ | Exhaustion  Physical  Mental | 264 (74.7)  306 (86.6) | Anxiety  Gender (physical)  Gender (mental) | β =0.232  X^2^ =0.273  X^2^ =0.134 | - | <.000  .601  .715 |
| Ha et al. (2022)  South Korea | RCT | March – June 2021 | 57 | Nurses | MFI |  |  | Mobile wellness program  *Pre-test*  *Post-test* | Intervention Group  92.63 (14.61)  87.37 (16.00) | 94.83 (13.90)  93.63 (19.00)  t =-1.06 | .295 |
| Haward et al. (2023)  India | Cross-sectional | May – August 2021 | 264 | Physicians  Nurses  Technicians | ADQ | Tiredness | - | PPE (doffing)  *Female*  *Male* | 127/184 (69.17)  102 (55.4)  82 (44.6) |  | .0.65 |
| Hawari et al. (2021)  Jordan | Cross-sectional  Survey | April – May 2020 | 937 | Nurses  Physicians  Technicians  Pharmacists | PROMIS | Fatigue | 319 (34.3) | Distress  Distress level  None  Low  Moderate  High | OR 2.40  0 (0)  34 (11.9)  91 (28.4)  196 (65.3) | - | <.001  <.001 |
| Hou et al. (2020)  China | Cross-sectional  Survey | March 2020 | 527 | Physician  Nurses | FS-14 | Fatigue | 298 (56.7) | Gender  Age (30-39 years)  Marital status  Education  Years worked  Technical title (senior, junior)  Self-efficacy  PTSD symptoms  Negative coping | -  -  -  -  -  Β =-0.392  r =.576  r =.143 | - | .233  .024  .233  .742  .398  .108  <.001  <.001  <.001 |
| Hou et al. (2022)  China | Cross-sectional  Survey | May 2020 | 507 | Nurses  Doctors | FS-14 | Fatigue | **M (SD)**  7.38 (4.43) | Re-experiencing  Avoidance  Hyperarousal  Negative coping style  Negative coping style & PTSS mediated by fatigue | r =0.467  r =0.535  r =0.593  β =0.17  ab = 0.09, SE= 0.03 |  | <.001  <.001  <.001  <.001  <.001 |
| Hwang et al. (2023)  South Korea | Cross-sectional  Survey | January, December 2020 | 1425 | HCW | FSS | Fatigue |  | **Chronic fatigue (FSS score = ≥3.22)**  Burnout (after outbreak)  Location (Ward, ER, ICU)  Number Covid19 patients cared for  Number of days caring for infected patients  **Physical fatigue**  Average time with infected patients per day | OR 3.94  OR 2.14  OR 0.82  OR 1.18  OR 1.34 |  | <.001  <.001  <.001  .034  <.001  <.001 |
| Jarrar et al. (2023)  Malaysia | Cross-sectional | October – December 2020 | 400 | Nurses | CFQ | Fatigue |  | Working hours (7, 8, 10, 12, >14) | F (4, 395) = 24.23 |  | <.001 |
| Jin et al. (2021)  China | Cross-sectional  Survey | March 2020 | 3912 | Doctors | NRS | Fatigue | 2049 (52.4)  **M (SD)**  4.15 (2.20) | Age  Gender  Junior clinicians  Working in tertiary hospital  Family/friends infected  Taking care of infected patients  Working experience  PHQ-9 total score ≥ 5  Quality of life | t =10.52  X^2^ = 1.32  OR 0.82  OR 1.23  OR 1.12  OR 1.16  OR 1.00  OR 7.40  F(1, 3,911) =283.75 |  | .54  .25  .045  .029  .494  .279  .902  <.001  <.001 |
| Karachalios et al. (2022)  Europe | Cross-sectional | December 2020 – May 2021 | 695 | Surgeons | ADQ | Fatigue | 183 (26) | - | - | - | - |
| Karimi et al. 2024)  Iran | RCT | February – September 2021 | 84 | Nurses | VAS-F | Fatigue | - | **Aromatherapy with Pelargonium graveolens**  Stage 1, Day 1 T2 v T1  T3 v T1  Stage 1, Day 2 T2 v T1  T3 v T1  Stage 1, Day 3 T2 v T1  T3 v T1 | **Mean Change (SD)**  -0.16 (1.2)  -0.38 (1.68)  -0.45 (1.01)  -0.57 (1.72)  -0.33 (0.9)  -0.69 (1.09) | 0.09 (0.57)  0.47 (0.96)  0.02 (0.68)  0.4 (0.88)  0.02 (0.51)  0.47 (0.8) | .208  .004  .014  .002  .029  .001 |
| Khalid et al. (2021)  Saudi Arabia | Cross-sectional | - | 102 | Nurses | ADQ | Exhaustion | 90 (88.2) | **M (SD)**  During MERS  During COVID | 1.51 (1)  2.06 (1) |  | <.001 |
| Kishi et al. (2022)  Japan | Cross-sectional | March 2021 | 896 | Nurses | AWAF | Fatigue | - | Caring for infected patients  Not caring for infected patients  **Burnout dimension**  Emotional exhaustion  Depersonalisation  Personal accomplishment  Social stress  Concerns for infection | β 0.445  β =0.089  r =.671  r =0.49  r =.234  r =.375  r =.384 |  | .022  NS  <.001  <.001  <.001  <.001  <.001 |
| Li, Guo et al. (2022)  China | Cross-sectional | December 2020 – May 2021 | 2968 | Nurses | CFQ | Fatigue | **M (SD)**  8.48 (3.40) | Presenteeism  Burnout  Health-related productivity loss  Mediation  *Fatigue mediated sickness presenteeism & productivity loss* | r =.201  r =.447  β =.248  - |  | <.001  <.001  <.001  <.001 |
| Li et al. (2021)  China | Cross-sectional | March – April 2020 | 139 | Doctors  Nurses  Others | ADQ | Fatigue | - | **PPE wearing**  *Yes*  *No*  *Duration of wearing PPE* | **M (SD)**  206.00 (39.73)  189.38 (44.60)  β =0.174 |  | .024 |
| Lie et al. (2021)  Norway | Cohort | May – July 2020 | 484 | Doctors  Nurses | ADQ | Exhaustion | 403 (87) | **Very exhausting**  *Professional cooperation*  *Social cooperation*  *Practical cooperation* | 81 (17)  55 (12)  58 (12) | -  -  - |  |
| Liu et al. (2023)  China | Cross-sectional  Survey | October – December 2021 | 270 | Nurses | MFI | Fatigue | **M (SD**)  45.94 (12.93) | Anxiety  Insomnia  Gender  Age  Daily working hours  PPE related symptoms  Fear of infection  Mediation  *Fear of infection on fatigue mediated by anxiety* | r =0.579  r =0.501  t =0.834  t =-0.515  t =3.568  t =5.451  t =-5.164  c=0.159 | - | < .01  < .01  .405  .607  <.001  <.001  <.001  < .01 |
| Ma et al. (2021)  China | Cross-sectional | November 2020 | 64 | Nurses | ADQ | Fatigue |  | **Physical fatigue**  Nursing Distance  *Title (Nurse, nursing assistant)*  *Work duration in Covid19*  Natural ventilation & light  *Title*  Spatial Privacy  *Title*  Psychological fatigue  Nursing Distance  *Title*  Spatial Congestion  *Work duration in Covid19*  Natural ventilation & light  *Title*  Spatial Privacy  *Title* | t =2.142  t =3.295  t =2.372  t =-1.97  t =4.276  t =2.456  t =-1.896  t =-2.89 |  | .034  .001  .031  .041  .007  .021  .031  .004 |
| Mahmood et al. (2020)  India | Cross-sectional  Survey | January – April 2020 | 240 | Doctors | ADQ | Fatigue | 115 (47.9) | Males  Females | 43 (30.2)  72 (73.4) | - | - |
| Mannocci et al. (2023)  Italy | Cross-sectional | August – September 2021 | 123 | Nurses | ADQ | Fatigue | **M (SD)**  2.94 (0.55) | Gender  Age  Working hours *(<7 hrs, >7 hrs)*  Working in Critical Area 1  Post-basic training (N/Y) | β =.177  β =.08  β =.247  β =.197  - | - | .039  .35  .004  .023  .03 |
| McAlonon et al. (2007)  Hong Kong | Prospective cohort  SARS | April – May 2003  April 2004 | 176 | HCW | ADQ | Fatigue | 90 (51.1) | Risk (isolation ward vs psychiatric unit)  SARS isolation ward  Psychiatric unit | X^2^ =37.9  106 (70.3)  70 (22.1) |  | <.05 |
| Mert et al. (2021)  Turkey | Cross-sectional  Survey | April – September 2020 | 729 | Nurses  Doctors  Others | WHOQOL-BREF-TR | Fatigue | - | PPE wearing  Physical health score  Psychological health score  Environmental wellbeing  Anxiety  Hopelessness | 201 (27.6)  r =−.263  r =−.124  r =−.272  r =.008  r =.210 |  | <.001  .001  <.001  .834  <.001 |
| Mohd Fauzi et al. (2020)  Malaysia | Cross-sectional  Survey | May 2020 | 1050 | Doctors | OFER-15 | **Fatigue**  Acute  Chronic | **M (SD)**  63.33 (19.02)  49.37 (24.47) | **Chronic fatigue**  Depression  Anxiety  Stress  Control  Mastery  Mental demand  Physical demand  Emotional demand  Temporal demand  Relaxation  Higher detachment | *r =.613*  *r =.527*  *r =.583*  *r = -.303*  *r = -.312*  *r =.239*  *r = .314*  β =3.64  β =1.02  β = 0.53  β =0.74 |  | <.01  <.01  <.01  <.01  <.01  <.01  <.01  .001  .006  .001  .001 |
| Mosheva et al. (2020)  Israel | Cross-sectional  Survey | March 2020 | 1106 | Physicians | ADQ | Exhaustion  Physical  Mental | 282 (25.5)  393 (35.5) | **Mental exhaustion**  *Anxiety* | β = 0.20 | - | <.001 |
| Nazario et al. (2023)  Brazil | Cross-sectional  Survey | July 2020 – February 2021 | 114 | Nurses | FAS | Fatigue | - | **Low vs High fatigue (score =>28)**  *Poor sleep quality*  *Work as cause of ill health*  *Medication due to work*  *Went to work sick*  *Has had work accident*  *Leave as a result of accident/illness*  *Health treatment due to work*  *Searched the occupational health department*  *Radiation exposure worrisome*  *Exposure to chemicals worrisome*  *Exposure to infectious diseases worrisome*  **Causes of discomfort**  *Noises/vibrations*  *Temperatures*  *Lighting*  *Air ventilation* | aOR 4.86  -  -  -  -  -  -  -  -  -  -  -  -  -  - | -  -  - | .008  .021  .015  .016  .656  .930  .095  .231  .277  .231  .417  .010  .379  .132  .693 |
| Ortabag et al.( 2022)  Turkey | Cross-sectional | May – June 2020 | 1414 | Nurses | OFER | Fatigue  Chronic  Acute | **M (SD)**  65.51 (25.83)  73.60 (21.90) | **Chronic fatigue**  *Gender*  *Age (19-25, 26-35, 36-58)*  *Marital status*  *No. of children (0, 1, 2-6)*  *Department (ICU, ER, other)*  *Professional experience (years)*  *Shift work (Y/N)*  *Night shifts (Y/N)*  *Night shift (more hours)*  *Caring for infected patients*  *Stress*  *Recovery dimension (OFER)*  *Acute fatigue* | -  -  -  -  -  -  -  -  -  -  r =.602  r =-.652  r =.717 |  | .009  .001  .04  .001  .017  .001  .028  .001  .001  .001  .001  .001  .001 |
| Patel et al. (2022)  USA | Prospective cohort | March, July, November 2020, March 2021 | 299 | Physicians | ADQ | Fatigue | - | Fatigue worse or much worse than before pandemic | X^2^ = 2.03 |  | .730 |
| Pearman et al. (2020)  USA | Cross-sectional  Survey | March – May 2020 | 90 | HCW | ADQ | Tiredness | **M (SD)**  2.93 (1.29) | - | - | **M (SD)**  1.85 (0.96) | <.001 |
| Rauch et al. (2021)  Italy | Randomised cross-over trial | September – October 2020 | 34 | EMS | ADQ | Fatigue |  | **M (SD)**  CPR Quality wearing PPE  *All compression parameters (depth, release, rate)* | 6.7 (1.8)  - | 5.0 (2.1)  - | <.001  1.00 |
| Sagherian et al. (2023)  USA | Cross-sectional  Survey | May – June 2020 | 587 | Nurses  Nursing assistant | OFER | Fatigue  Acute  Chronic | **M (SD)**  77.06 (17.62)  60.39 (24.06) | Chronic fatigue  *Cared for infected patients*  *Did not care for infected patients*  *Years of experience*  *Work status (FT/PT)*  *Working hours (< 40 v > 40*  *Shift length (extended v traditional)*  *Shift type (day, night, rotating)*  *30-min breaks* | **M(SD)**  60.39 (24.06)  54.27(25.38)  F=0.27  T=2.44  T=-2.87  T=-0.35  T=1.76  F=9.38 |  | <.001  .845  .015  .004  .726  .080  <.001 |
| Santric-Milicevic et al. (2024)  Serbia | Cross-sectional | December 2021 | 4210 | Nurses | ADQ | Exhaustion | 1998 (48.1) | Increased tension, stress & pressure  Exhaustion due to volume of work  Exhaustion due to PPE wearing | OR 1.13  OR 1.41 |  | .161  <.001 |
| Serin & Calgar (2021)  USA | Quasi-experiment | NR | 48 | Physicians  Paramedics | VAS-F | Fatigue | - | CPR Parameters using PPE  Surgical Mask  FFR  Half-face  No-mask  Surgical compared to no mask | **Median (IQR)**  5 (5/6)  7 (7/8)  9 (9/10)  5 (4/6)  - |  | <.001  .150 |
| Sharma et al. (2022)  India | Cross-sectional  Survey | June 2020 | 2334 | Nurses  Doctors | ADQ | Exhaustion | 619 (26.5) | Gender  Age  Profession (Doctor)  Higher education level  Marital status (divorced or widowed)  Pre-existing comorbidities  Posted in the COVID19 outpatient department  Working in states with > 50,000 cases | **-**  **-**  OR 3.1  OR 1.4  OR 2.5  OR 1.6  OR 1.6  OR 3.1 |  | .005  .760  .001  .005  .011  .004  .015  <.001 |
| Sikaras et al. (2023)  Greece | Cross-sectional | November – December 2021 | 404 | Nurses | FAS | Fatigue | **M (SD)**  24.08 (7.35) | Gender  *Women*  *Men*  Trait Anxiety  Depression  State anxiety | 24.66 (7.20)  21.25 (7.43)  β = 0.297  β = 0.373  β = 0.180 |  | <.01  <.01  <.01  <.01 |
| Song et al. (2023)  South Korea | Cross-sectional | February – March 2021 | 140 | Nurses | ADQ | Fatigue | **M (SD)**  3.47 (0.55) | Empowerment scores  Depression | r =–0.07  β =0.25 | - | .388  .001 |
| Soylu et al. (2023)  USA | Cross-sectional  Survey | November – December 2020 | 86 | Nurses | MPSTEFS | Trait fatigue  Trait energy | - | Depression  Anxiety  Loneliness | β =0.93  β =0.96  r =.129 |  | <.001  <.004  .236 |
| Stecca et al. (2022)  Canada | Cross-sectional  Survey | July – August 2020 | 24 | Doctors | ADQ | Fatigue/  Low energy | 12 (50) | - | - | - | - |
| Su et al. (2021)  China | RCT | May – June 2021 | 68 | Doctors  Nurses | ADQ | Fatigue | - | Mask conditions  N95 Respirator mask  Surgical mask | 9/34 (27)  0/34 |  | .001 |
| Sun et al. (2023)  China | Cross-sectional  Survey | December 2020 – January 2021 | 374 | Nurses | 3D-WFI | Fatigue  Physical  Mental  Emotional | 358 (95.7)  369 (98.6)  275 (73.5) | Burnout  Job satisfaction  Turnover intention | β =9.28  β =−8.03  β =8.49 |  | <.01  <.01  <.01 |
| Swaminathan et al. (2022)  UK | Cross-sectional | July – August 2020 | 72 | Surgeons  Anaesthetists  Nurses  Others | VAS | Fatigue | - | PPE | 51 (70.8) | - | - |
| Tabah et al. (2020)  Worldwide | Cross -Sectional  Survey | April 2020 | 2711 | Physician  Nurses  Allied HCW | ADQ | Exhaustion | - | PPE Hours  Every 1 hr increase in PPE use | 492 (20)  OR 1.11 |  | <.001 |
| Tang et al. (2022)  China | Cross-sectional  Survey | August – September 2021 | 233 | Nurses  Doctors  Technicians  Others | FSAS | Fatigue | **M (SD)**  24.10 (24.38) | GAD-7 Score  PHQ-9 score  Sleep duration  Sleep quality (good)  Title (junior)  Physical fatigue  *Anxiety*  *Depression*  Mental fatigue  *Anxiety*  *Depression*  Consequences of fatigue  *Anxiety*  *Depression*  Fatigue response to sleep/rest  *Anxiety*  *Depression*  Severity of fatigue  *Anxiety*  *Depression* | β =0.019  β =0.529  β =0.039  β =0.018  β =0.061  OR 0.99  OR 1.01  OR 1.01  OR 1.09  OR 1.05  OR 1.02  OR 1.01  OR 1.04  OR 1.00  OR 1.00 |  | .755  .000  .826  .964  .897  .968  .594  .543  .005  .061  .572  .079  .008  .293  .577 |
| Teng et al. (2020)  China | Cross-sectional  Survey | NR | 398 | HCW | FSAS | Fatigue | **M (SD)**  16.4 (19.2) | - | - |  | - |
| Teo et al. (2021)  Singapore | Cross-sectional  Survey | May – June 2020 | 122 | Laboratory | ADQ | Exhaustion | - | Increased workload  Depression | OR 3.4  OR 6.1 | - | .01  .02 |
| Thiagarajan et al. (2021)  India | Cross-sectional  Survey | August – September 2020 | 342 | Surgeons | ADQ | Fatigue | - | PPE wearing  *Poor visibility*  *Fogging*  *Uncomfortable*  *Incompatible with loupes*  *Skin issues*  *Communication problem*  *Dryness of mouth*  *Breathing/suffocation*  *Headache* | 236 (69)  -  -  -  -  -  -  -  OR 4.24  OR 11.34 |  | <.001  .139  .05  .34  .09  .315  .02  <.001  <.001 |
| Vamvakas et al. (2022)  Greece | Cross-sectional | - | 147 | Nurses  Doctors  Others | ADQ | Exhaustion | - | Higher exhaustion  *Anxiety*  *Depression* | **M (SD)**  88.06  52.03 |  | .023  .003 |
| Vanhaecht et al. (2021)  Belgium | Cross-sectional  Survey | April – May 2020 | 4509 | Paramedics  Nurses  Doctors  Others | ADQ | Fatigue | - | **During vs before Covid19**  Age  Gender  *Female*  *Male*  Profession  *Paramedic*  *Nurses*  *Doctors*  *Between professions*  Care sector  *Hospital*  *Primary care*  *Residential healthcare*  *Mental healthcare*  *Between sectors* | -  OR 6.30  OR 5.15  OR 5.15  OR 8.64  OR 4.42  OR 7.19  OR 5.57  OR 7.94  OR 6.81  - | -  -  -  -  -  -  -  -  - | 0.44  0.64  <.01  <.01  <.01  <.001  <.01  <.01  <.01  <.01  <.001 |
| van Roekel et al. (2021)  Netherlands | Cross-sectional  Survey | May – June 2020 | 7208 | HCW | ADQ | Exhaustion | **-** | **Physical exhaustion**  *Gender*  *Age (>55 years)*  *Living alone*  *Leadership role*  *Sufficient PPE*  *Contact with patients*  **Mental exhaustion**  *Age (<36 years)*  *Living alone*  *Sufficient PPE* | β =-0.05  β =0.07  β =0.05  β =-0.04  β =0.19  d=.223  β =0.06  β =0.05  β =0.23 |  | <.05  <.05  <.05  <.05  <.05  <.01  <.05  <.05  <.05 |
| Wang et al. (2021)  China | Cross-sectional | February 2020 | 115 | Doctors  Nurses | MFI-20 | Fatigue | **M (SD)**  51.02 (10.46) | Age  Gender  Technical Title  Duration in Wuhan (>25 days)  Profession (doctor, nurse)  Marital status  Work experience (>10 years)  Department (ICU, isolation)  Stress level  Current health status  Education  Resilience  Anxiety  Physical burden  **Mediation**  Resilience – Fatigue – Anxiety  Resilience – Fatigue - Physical burden  Fatigue - Physical burden -Anxiety | **T/F Value**  0.066  2.142  0.069  -3.295  1.195  0.170  0.554  1.822  4.276  4.978  -1.266  β =-0.52  β =0.12  β =0.65  β =-0.305  β = 0.276  β =0.174 |  | .936  .034  .945  .001  .234  .866  .581  .071  .007  .003  .203  .000  .166  .000  .039  .020  .153 |
| Wu et al. (2021)  China | Cross-sectional | March 2020 | 1337 | Nurses | FS-14 | - | **M (SD)** | Class 3 vs 1*  Class 3 vs 2*  Class 2 vs 1  Physical fatigue between 3 classes  Mental fatigue between 3 classes  Total FS-14 between 3 classes | β =0.200  β =0.428  β =−0.047  -  - |  | .541  .033  .735  .028  .075  .045 |
| Xu et al. (2024)  China | Cross-sectional | June – July 2020 | 471 | Nurses | 3D-WFI |  |  | **Physical fatigue**  Strength  Optimism  Tenacity  Psychological resilience  **Mental fatigue**  Strength  Optimism  Tenacity  Psychological resilience  **Emotional fatigue**  Strength  Optimism  Tenacity  Psychological resilience  **Physical fatigue**  **Profile 1 vs 2  Profile 1 vs 3  Profile 2 vs 3  **Mental fatigue**  Profile 1 vs 2  Profile 1 vs 3  Profile 2 vs 3  **Emotional fatigue**  Profile 1 vs 2  Profile 1 vs 3  Profile 2 vs 3 | r =-0.410  r =-0.229  r =-0.363  r =−0.397  r =-0.436  r =-0.236  r =-0.394  r =−0.426  r =-0.451  r =-0.241  r =-0.374  r =−0.420  X^2^ =15.318  X^2^ =62.424  X^2^ =21.755  X^2^ =26.436  X^2^ =77.060  X^2^ = 18.260  X^2^ =33.865  X^2^ =76.362  X^2^ =12.129 |  | <.01  <.01  <.01  <.01  <.01  <.01  <.01  <.01  <.01  <.01  <.01  <.01  <.001  <.001  <.001  <.001  <.001  <.001  <.001  <.001  <.001 |
| Yanez Benitez et al. (2020)  Worldwide | Cross-sectional  Survey | April 2020 | 134 | Surgeons | ADQ | Fatigue | - | PPE | 110 (82) | - | - |
| Yin et al (2023)  China | Cross-sectional | December 2021 | 4901 | Doctors  Nurses  Pharmacists | ADQ | Fatigue | 3307 (67.4) | **Severe fatigue**  *Depression*  *Anxiety*  Between doctors, nurses, others | OR 2.55  OR 4.15  **-** |  | <.001  <.001  <.001 |
| Yucel et al. (2024)  Turkey | Cross-sectional | February, March 2021 | 793 | Doctors  Nurses  EMT | OFER | Fatigue  Chronic  Acute | 60.84 (33.87)  68.97 (23.3) | **Chronic fatigue**  Depression  Anxiety  Stress  Intolerance of uncertainty  Gender  Marital status  Having children  Chronic disease in self/family  PPE (sufficient/insufficient)  Working in contact tracing  Disruption of routine operations  Patients missing follow-up appts  Increase in demand for primary care services | r =.522  r =.435  r =.507  r =.393  OR .052  OR 1.21  OR 0.89  OR 0.60  OR 1.65  OR 0.97  OR 0.69  OR 0.75  OR 0.83 |  | <.01  <.01  <.01  <.01  .026  .442  .644  .001  .001  .915  .094  .190  .412 |
| Zhan et al. (2020)  China | Cross-sectional | March 2020 | 1794 | Nurses | FS-14 | Fatigue | 609 (33.9) | Insomnia | β =0.379 |  | <.001 |
| Zhang, Hu et al. (2021)  China | Cross-sectional | March 2020 | 249 | Physicians  Nurses | FS-14 | Fatigue | **M (IQR)**  3 (1,6) | P**oor sleep vs good sleep quality**  *Mental fatigue*  *Physical fatigue*  Severe fatigue | **X^2/^/Z**  4.228  6.436  OR 1.51 |  | <.01  <.01  .015 |
| Zhang, Liu et al. (2023)  China | Cross-sectional  Survey | November 2021 | 270 | Nurses | MFI-20 | Fatigue | M(SD)  45.93 (12.92) | Transition shock  Anxiety  Cooperation  Daily working hours | β=0.687  β=0.757  β=−0.262  β=−0.007 |  | < 0.001  < 0.001  < 0.001  > 0.05 |
| Zhang, Li et al. (2022)  China | Cross-sectional | February – March 2020 | 810 | Doctors  Nurses | FSS | Fatigue | 602 (74.3) | Profession (nurse)  Gender  Exposure to high-risk areas  Designated hospital  Age  Location (Wuhan, Hubei, outside Hubei) | aOR 0.54  aOR 1.83  aOR 3.87  Z=-2.613  H=5.178  H=19.653 |  | .022  .028  <.001  .009  .270  .000 |
| Zhang, Xu et al. (2021)  China | Cross-sectional | March - April 2020 | 402 | Nurses | 3D-WFI | Fatigue | M (SD)  Mental fatigue  3.58 (1.09)  Physical fatigue 3.19 (1.24)  Emotional fatigue 2.78 (1.20) | Resilience  Mental fatigue  Physical fatigue  Emotional fatigue  Perceived organisational support (POS)  Mental fatigue  Physical fatigue  Emotional fatigue  Resilience mediated between POS and mental, physical and emotional fatigue | β = −0.274  β = −0.322  β = −0.343  β = −0.264  β = −0.283  β = −0.285 | < 0.001  < 0.001  < 0.001  < 0.001  < 0.001  < 0.001  <0.001 |  |
| Zou et al. (2021)  China | Cross-sectional  Survey | February - March 2020 | 3557 | Doctors  Nurses  Laboratory | ADQ | Fatigue | 64.5 (2299) | Gender (Female)  Frontline  Disease control HCW  Depression  Anxiety  Insomnia  Social support  Education (bachelor’s degree)  Divorced/widowed  Profession  Hours per day (12 hours)  Sleep  <5 hours  5 hours  6 hours  >7 hours | OR 0.77  OR 1.83  OR 3.59  OR 2.02  OR 1.52  OR 2.45  OR 2.45  OR 1.35  OR 1.36  OR 7.26  OR 7.80  OR 5.32  OR 3.05  OR 1.88 |  |  |
| Zhou et al. (2021)  China | Cross-sectional | February – April 2020 | 494 | HCW | NRS | Fatigue | - | Good psychiatric status vs Poor psychiatric status  Poor psychiatric status | X^2^ =−8.188  OR 1.266 |  | <.001  .003 |

aOR= adjusted odds ratio; OR = odds ratio; r = correlation; X^2^ = chi square; M = mean; IQR = interquartile range; 3D-WFI= Work Fatigue Inventory; ADQ=author designed questionnaire; CFQ=Chalder Fatigue Questionnaire; FAS=Fatigue Assessment Scale; FFR= filtering face-piece respirator; FS-14=14-item Fatigue Scale; FSAS=Fatigue Self-Assessment Scale; FSS=Fatigue Severity Scale; HCW=healthcare workers; HRZ=High risk zone; MAF= Multidimensional Assessment of Fatigue; MFI=Multidimensional Fatigue Scale; MPSTEFS=Mental and Physical State and Trait Energy and Fatigue Scale; MTC-Q1=Music Care Team-Q1; NRS=Numeric Rating Scale; OFER= Occupational Fatigue Exhaustion Recovery Scale; POMS=Profile of Mood States; AWAF=20-item Self-Diagnosis Check List for Assessment of Workers’ Accumulated Fatigue; VAS-F=Visual analogue scale (Fatigue)

*Class 1: low workload perception & high self-evaluation group; Class 2: medium workload perception & medium self-evaluation group; Class 3: high workload perception & low self-evaluation group

** Profile 1: low psychological resilience; Profile 2: medium psychological resilience; Profile 3: high psychological resilience
